# iPSC–derived retinal pigmented epithelial cells from patients with macular telangiectasia show decreased mitochondrial function

**DOI:** 10.1172/JCI163771

**Published:** 2023-05-01

**Authors:** Kevin T. Eade, Brendan Robert E. Ansell, Sarah Giles, Regis Fallon, Sarah Harkins-Perry, Takayuki Nagasaki, Simone Tzaridis, Martina Wallace, Elizabeth A. Mills, Samaneh Farashi, Alec Johnson, Lydia Sauer, Barbara Hart, M. Elena Diaz-Rubio, Melanie Bahlo, Christian Metallo, Rando Allikmets, Marin L. Gantner, Paul S. Bernstein, Martin Friedlander

**Affiliations:** 1 The Lowy Medical Research Institute, La Jolla, California, USA.; 2 Department of Molecular Medicine, The Scripps Research Institute (TSRI), La Jolla, California, USA.; 3 Population Health and Immunity Division, Walter and Eliza Hall Institute of Medical Research, Parkville, Victoria, Australia.; 4 Department of Medical Biology, The University of Melbourne, Parkville, Victoria, Australia.; 5 Department of Ophthalmology and; 6 Department of Pathology and Cell Biology, Columbia University, New York, New York, USA.; 7 Institute of Food and Health, School of Agriculture and Food Science, University College Dublin, Dublin, Ireland.; 8 Moran Eye Center, University of Utah School of Medicine, Salt Lake City, Utah, USA.; 9 Molecular and Cell Biology Laboratory, Salk Institute for Biological Studies, La Jolla, California, USA.; 10 Department of Bioengineering, University of California San Diego, La Jolla, California, USA.

**Keywords:** Ophthalmology, Stem cells, Mitochondria, Retinopathy, iPS cells

## Abstract

Patient-derived induced pluripotent stem cells (iPSCs) provide a powerful tool for identifying cellular and molecular mechanisms of disease. Macular telangiectasia type 2 (MacTel) is a rare, late-onset degenerative retinal disease with an extremely heterogeneous genetic architecture, lending itself to the use of iPSCs. Whole-exome sequencing screens and pedigree analyses have identified rare causative mutations that account for less than 5% of cases. Metabolomic surveys of patient populations and GWAS have linked MacTel to decreased circulating levels of serine and elevated levels of neurotoxic 1-deoxysphingolipids (1-dSLs). However, retina-specific, disease-contributing factors have yet to be identified. Here, we used iPSC-differentiated retinal pigmented epithelial (iRPE) cells derived from donors with or without MacTel to screen for novel cell-intrinsic pathological mechanisms. We show that MacTel iRPE cells mimicked the low serine levels observed in serum from patients with MacTel. Through RNA-Seq and gene set enrichment pathway analysis, we determined that MacTel iRPE cells are enriched in cellular stress pathways and dysregulation of central carbon metabolism. Using respirometry and mitochondrial stress testing, we functionally validated that MacTel iRPE cells had a reduction in mitochondrial function that was independent of defects in serine biosynthesis and 1-dSL accumulation. Thus, we identified phenotypes that may constitute alternative disease mechanisms beyond the known serine/sphingolipid pathway.

## Introduction

Complex diseases of aging provide unique challenges for the identification of specific disease-causing genetic variants and associated cellular dysfunctions using traditional genetic approaches. Disease modeling using iPSCs is a powerful tool for identifying such cell-specific disease mechanisms, with a significant advantage in that it requires relatively few patients (1). iPSCs can faithfully recapitulate donor-specific gene expression and disease-specific phenotypes, allowing for the study of complex human traits (2–4). Despite clonal heterogeneity of iPSCs, their genetic background is the main contributing factor to differences in gene expression and cellular function (4). Donor-derived iPSC disease models have been successful in illuminating mechanisms of complex multivariant and even late-onset diseases such as Alzheimer’s and Parkinson’s (5, 6), and recent advancements in the differentiation of iPSCs into specific tissues have been particularly advantageous for the study of metabolic disorders with a tissue-specific manifestation (7).

One disease that lends itself particularly well to this approach is macular telangiectasia type 2 (MacTel), a relatively uncommon retinal degenerative disease with an estimated prevalence of 1:1,000 to 1:22,000 people that leads to progressive loss of central vision (8–10). MacTel has a complex genetic architecture, as indicated by the analysis of extended families with multiple affected individuals, linkage studies, GWAS, and the identification of multiple rare causative variants (11–15). Recently we have shown that MacTel is linked to deficiencies in serine and glycine metabolism, which drives a concomitant increase in a neurotoxic, atypical lipid species, 1-deoxysphingolipids (1-dSLs) (13–15).

MacTel patient populations have reduced circulating levels of serine and glycine and elevated levels of 1-dSLs, which are linked to the onset and severity of disease (13, 14, 16). We have identified multiple causal variants for MacTel in genes that drive serine and sphingolipid metabolism: variants in the serine palmitoyltransferase (SPT) enzymatic complex that initiates de novo ceramide synthesis from serine and palmitate, and variants in phosphoglycerate dehydrogenase (PHGDH), the rate-limiting enzyme in the serine biosynthesis pathway (14, 15). Gain-of-function mutations in the SPT complex, SPTLC1 (p.Cys133Tyr) and SPTLC2 (p.Ser384Phe), directly elevate the synthesis of 1-dSLs in patients by preferentially condensing alanine instead of serine with palmitoyl–coenzyme A (palmityol-CoA) to generate the initial sphingoid base. These mutations are also causative for a severe peripheral neuropathy, hereditary sensory and autonomic neuropathy type 1 (HSAN1); however, they are extremely rare in patients with MacTel, accounting for less than 0.1% of the disease load (14, 15). Loss-of-function mutations in *PHGDH* decrease serine biosynthesis, which subsequently increases 1-dSL levels indirectly by reducing serine availability for SPT (15). Although *PHGDH* variants are the most abundant coding mutations found in MacTel, they collectively account for only 3.2% of the disease load (15). GWAS analyses have also identified several common genetic loci associated with MacTel, some of which overlap with genes linked to serine and sphingolipid biosynthesis, including *PHGDH* (13, 17). Determining additional pathological mechanisms through the identification and characterization of genetic factors in MacTel is particularly difficult, as coding mutations are exceptionally rare (15), and determining the function of noncoding, disease-linked GWAS loci is uncommon (18). In this study, we characterize shared cellular defects in donor-derived induced pluripotent stem cells (iPSCs) to identify what we believe to be a novel disease mechanism in MacTel.

Successful identification of disease phenotypes in differentiated iPSCs depends on the appropriate selection of disease-relevant cell types and tissues. The molecular and cellular bases of MacTel remain unclear, as much of the metabolic data linking serine metabolism to MacTel have measured systemic levels of metabolites in patients with MacTel (13, 14). Clinical observations in late stages of MacTel retinopathy show a loss of photoreceptors and Müller glia within the macula, accompanied by a pan-retinal loss of structure and function in the retinal pigmented epithelium (RPE) (19, 20). This subclinical cellular defect in RPE is not restricted to the MacTel zone, indicating that RPE pathology in MacTel is not secondary to the degeneration occurring in the central retina and may be a primary defect contributing to disease. RPE cells are specialized support cells critical for the health of the outer retina. They are situated in the interface between photoreceptors and the choriocapillaris and form the outer blood-retina barrier (21, 22). These cells provide metabolic support to the outer retina by phagocytosing photoreceptor outer segments for lipid recycling, as well as transporting and synthesizing nutrients and key metabolites. In the outer retina, RPE cells are a primary source of serine and glycine, which they synthesize and secrete to photoreceptors (23). MacTel-specific mutations in *PHGDH* and *SPTLC1* directly affect the RPE, causing decreased serine and increased 1-dSL synthesis (15). Given the central role RPE plays in disease progression, the independent pathology of these cells, and their expression of disease-linked genes, it is likely that patients with MacTel have RPE cells with cell-autonomous defects in addition to nonautonomous effects.

Here, we generated iPSC-derived RPE (iRPE) from patients with MacTel and control, non-MacTel donors with diverse genetic backgrounds to screen for functional defects associated with a disease state in MacTel. We validated that iRPE cells from MacTel donors exhibited lower levels of serine and glycine as well as an upregulation of serine/glycine biosynthesis genes, consistent with known disease phenotypes in patients. We performed RNA-Seq and gene set enrichment analysis (GSEA) on iRPE, identifying an induction of stress pathways and potential defects in oxidative phosphorylation (OXPHOS) in MacTel donor cells. In a subsequent functional analysis, we observe that MacTel iRPE cells had reduced mitochondrial function. Finally, CRISPR editing to correct a MacTel-linked PHGDH variant in 1 donor cell line showed that the observed mitochondrial defects were not dependent on serine biosynthesis. Thus, we established donor-derived iRPE as a model for molecular mechanisms of MacTel pathology and identified what we believe to be a new cell-intrinsic disease phenotype in MacTel that is independent of the known serine/sphingolipid mechanism.

## Results

### Donor patient cohort.

**Figure 1:**
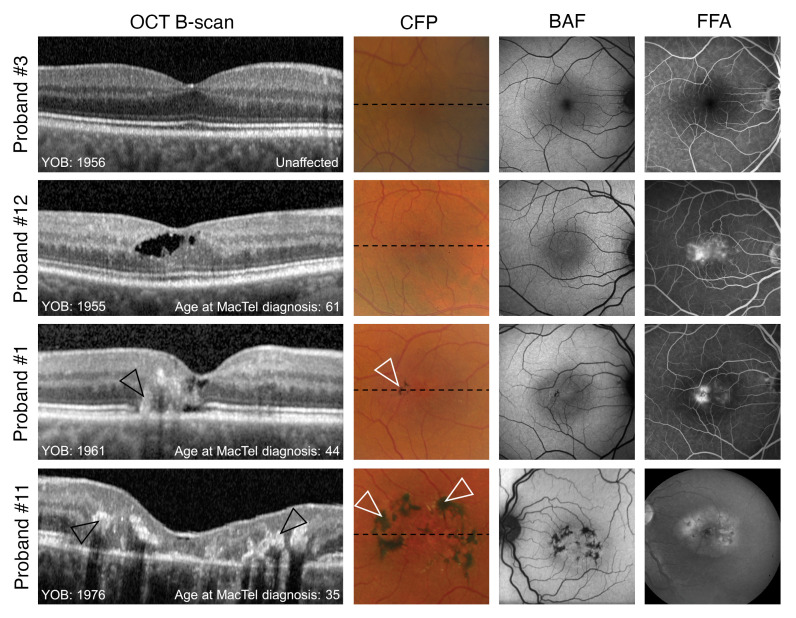
Multimodal retinal images in 4 representative eyes from 4 probands illustrating different stages of disease severity in MacTel and a healthy control proband. Probands 12, 1, and 11 show characteristic findings of MacTel, including increased central blue light autofluorescence (BAF) signaling and vascular leakage on fundus fluorescein angiography (FFA). Findings associated with more advanced disease stages, including a disruption of outer retinal layers, retinal hyper-reflectivity (black arrowheads), and perivascular pigment plaques (white arrowheads) can be found in proband 1 and are most pronounced in proband 11. Note the unusually young age, age of onset, and advanced disease state for proband 11. CFP, color fundus photography; OCT, optical coherence tomography; YOB, year of birth. Black dotted lines indicate the position of the respective B scans on OCT.

We collected PBMCs from MacTel (*n* = 9) and non-MacTel control (*n* = 5) donors at 2 clinical sites. From available clinical samples, MacTel and control cohorts were balanced for age and sex as much as possible (Table 1). Affected donors were on average 4.8 years younger than unaffected donors. Our studied included 3 affected males, 6 affected females, 3 unaffected males, and 2 unaffected females. The MacTel donors were representative of typical MacTel cases (Figure 1). They were first diagnosed with MacTel at an average age of 53 years, which is slightly younger than the average age, 57 years, for MacTel diagnosis (24). The control donors were selected from unaffected family members and were on average 66 years old at the time of their last ophthalmological exam confirming the absence of MacTel. Neuropathy indicative of HSAN1 was not detected in any donor. All patients were screened for known MacTel-linked protein-coding variants (Table 1 and Supplemental Table 1; supplemental material available online with this article; https://doi.org/10.1172/JCI163771DS1). We identified a heterozygous, disease-associated c.628G>T (p.G228W) *PHGDH* variant in an affected individual (donor 11), who presented with an early-onset and rapidly progressing form of MacTel (Supplemental Figure 1A). She was diagnosed at age 35 and was legally blind within 5 years of the diagnosis. Examinations of family members resulted in a diagnosis of MacTel in donor 11’s daughter at age 22 (Supplemental Figure 1B). The intergenerational, rapidly progressing, and early-onset form of MacTel in donor 11 suggests that these individuals have a severe form of the disease.

### Generation of iRPE cells from donor PBMCs.

**Figure 2:**
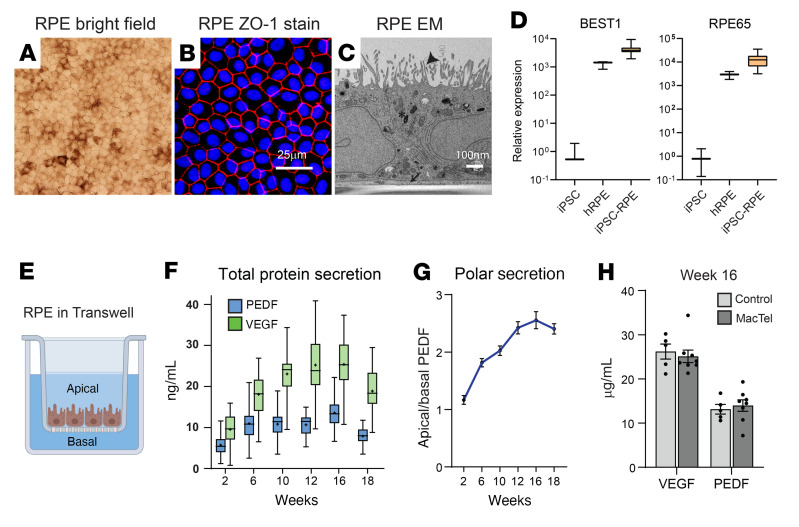
Donor iPSC–derived RPE cells are functional RPE cells. (**A**) Representative bright-field image of iRPE cells showing hexagonal morphology and pigmentation. Original magnification is ×20.(**B**) Representative confocal image of iRPE cells showing ZO-1 (red) and DAPI (blue) staining. (**C**) Representative transmission electron microscopy image of a cross-section of iRPE showing apical microvilli (arrowhead), basement membrane (arrow), and pigment granules (asterisk). (**A**–**C**) Images are from iRPE from MacTel-affected donor 9. (**D**) Relative expression levels of the RPE-specific genes *BEST1* and *RPE65* in iPSCs (*n* = 3 clones), human fetal RPE cells (*n* = 3 replicates), and donor-derived iRPE cells (*n* = 38 clones). (**E**) Cartoon representation of iRPE cells cultured in a Transwell with separate apical and basal media chambers. (**F**) Total protein secretion of VEGF and PEDF from iRPE cells at different time points over a 24-hour period. Data are shown as maximal to minimal box plots, with the line as the median and “+” as the mean. *n* = 35 clones. (**G**) Polar secretion of PEDF from iRPE at different time points. *n* = 35 clones. (**H**) Total VEGF and PEDF levels at 16 weeks in MacTel and control iRPE. VEGF, *P* = 0.74 and PEDF *P* = 0.85, using mixed linear modeling. Control, *n* = 5 donors; MacTel, *n* = 8 donors. Each individual donor is represented by the average of at least 2 independent clones. All data are represented as the mean ± SEM. Scale bars: 25 μm (**A** and **B**) and 100 nm (**C**).

To develop a model in which to screen for disease-associated cellular dysfunction in MacTel RPE, we derived a library of donor iPSCs and then generated homogenous monocultures of iRPE cells. Multiple independently derived iPSC clones were generated from each donor’s PBMCs. Each clone was validated for pluripotency markers and screened for chromosomal abnormalities. Donor iPSCs were differentiated into RPE cells through directed differentiation. iRPE monocultures formed hexagonal pigmented monolayers (Figure 2A) and displayed characteristic morphological RPE features, including apical microvilli, a basement membrane, and tight junctions (Figure 2, B and C, and Supplemental Figure 2A). iRPE cells strongly expressed the RPE-specific markers BEST1, RPE65, and MERTK compared with undifferentiated iPSCs and cultured human fetal RPE cells (Figure 2D and Supplemental Figure 2B). We detected no difference in expression for these markers between control and MacTel iRPE cells (Supplemental Figure 2C). Following initial differentiation, iRPE cultures require additional postmitotic maturation in confluent monolayers to obtain proper gene expression, morphology, and function. Since iRPE cells differentiated from different iPSC clones may mature at variable rates, we determined a time point at which all iRPE clones reached a stable mature state, ensuring that we compared iRPE function rather than differentiation potential. Mature RPE, both in vitro and in vivo, polarize so that they secrete pigment epithelium-derived factor (PEDF) apically and VEGF basally (21). To validate both the RPE-specific function of differentiated iRPE cells and select the time point of collective maturation, we measured protein levels of secreted VEGF and PEDF using the Mesoscale immunoassay in control and MacTel iRPE cells cultured in Transwells over the course of 18 weeks (Figure 2, E–H). Collectively, iRPE cells showed a progressive increase in VEGF and PEDF secretion levels out to 16 weeks of maturation (Figure 2F) as well as maximum polarization in secretion at week 16 (Figure 2G). This time point of maturation is similar to that seen in previous work showing that iRPE cells require up to 18 weeks to functionally mature (25). Between the patient with MacTel and the control iRPE cohorts, we observed no differences in the abundance of secreted VEGF or PEDF (Figure 2H). On the basis of these findings, we selected 16 weeks as the defining time point for functional assays in subsequent experiments. Since assay batch effects can provide greater variability than the genetic background of the iPSC donor (2), all experiments were performed from iRPE cells cultured concurrently.

### MacTel iRPE cells have normal phagocytosis.

**Figure 3:**
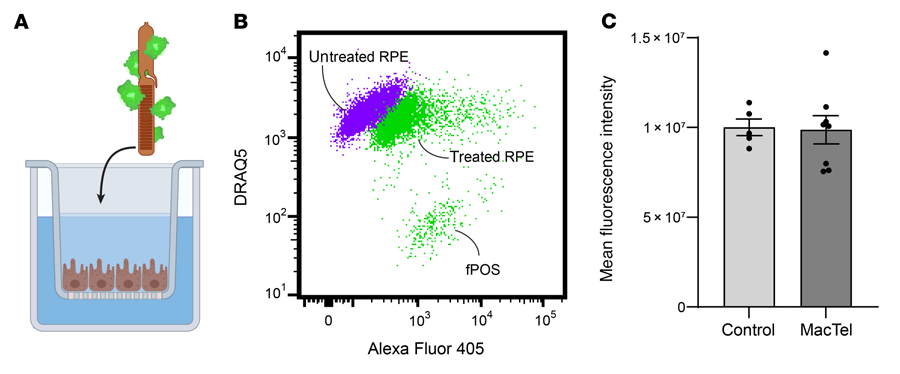
Phagocytosis in donor-derived iRPE cells. (**A**) Schematic illustrating the addition of fPOSs to the iRPE culture. (**B**) Representative plot of flow cytometry gating showing the shift in fluorescence from untreated iRPE cells (purple) and iRPE cells phagocytosing POSs for 5 hours (green). (**C**) MFI of control (*n* = 5 donors) and MacTel (*n* = 8 donors) iRPE cells. Data are represented as the mean of the donors ± SEM. Each individual donor represents the average of at least 2 independent clones run in duplicate. Statistical significance was determined using mixed linear modeling.

Clinical observations in late-stage MacTel show an accumulation of subretinal cellular debris between photoreceptor outer segments and RPE throughout the entire retina, suggesting that there may be a pan-retinal RPE cell phagocytosis defect (20). We next tested whether iRPE cells derived from MacTel donors (a) were functionally mature, phagocytosing RPE cells and (b) had an inherent phagocytosis defect by assaying their ability to phagocytose fluorescence-conjugated porcine outer segments (fPOSs) as previously described (24) (Figure 3, A and B). Following a 5-hour period of incubation with fPOSs, we observed that iRPE cells had robust phagocytic activity. We observed no differences in fPOS uptake between iRPE cells from the MacTel or control donor cohorts (Figure 3C).

### MacTel iRPE cells have decreased serine and glycine.

**Figure 4:**
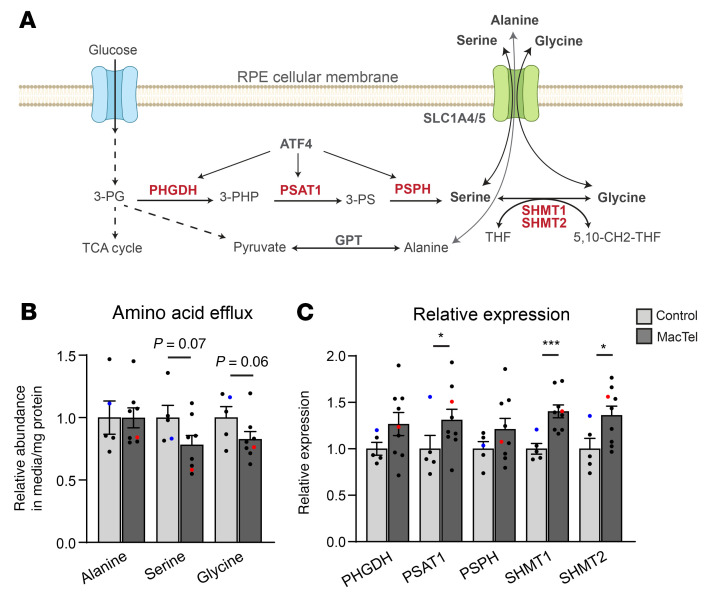
MacTel iRPE cells have reduced serine and glycine. (**A**) Schematic illustrating serine, glycine, and alanine biosynthesis from central carbon metabolism in RPE. (**B**) Relative abundance of alanine, serine, and glycine secreted into the media from control (*n* = 5 donors) and MacTel (*n* = 8 donors) cells. Data are represented as the mean of the donors ± SEM. Each individual donor represents the average of at least 2 independent clones run in triplicate. (**C**) Relative expression of serine/glycine biosynthesis enzyme genes in control (*n* = 5 donors) and MacTel (*n* = 9 donors) cells in amino acid–depleted media. Donor 11 (heterozygous for the p.G228W PHGDH variant) is represented by a red dot. Donor 6 (heterozygous for the p.T152I PSPH variant) is represented by a blue dot. Data are represented as the mean of the donors ± SEM. Each individual donor is the average of at least 2 independent clones run in duplicate. *P < 0.05 and ***P < 0.001, by mixed linear modeling.

Metabolomic screens of patient serum have shown that serine and glycine are the most significantly dysregulated metabolites in patients with MacTel (13, 26). RPE cells are a major source of serine and glycine in the retina. Through the serine biosynthesis pathway, RPE cells convert glucose to serine and glycine and secrete these metabolites from their apical side toward the retina (15, 23) (Figure 4A). We have recently reported that rare MacTel-linked coding variants in *PHGDH* reduced the synthesis and secretion of serine and glycine in iRPE, with the largest decrease observed in the amino acids secreted into the media (15). Therefore, to detect differences in serine and glycine abundance in donor iRPE, we measured the secreted amino acid abundance (Figure 4). We found a 22% mean reduction in serine secretion and a 17% mean reduction in glycine secretion compared with controls (Figure 4B), similar to the reduction in plasma levels of serine and glycine observed in patients with MacTel (13, 14). However, the reduced abundance of serine and glycine in MacTel iRPE only had borderline *P* values of 0.068 and 0.056, respectively (Figure 4B). In a repeat experiment, we observed similar trends, and the decrease in glycine secretion passed the significance threshold (*P* = 0.04) (Supplemental Figure 3A). One patient, donor 11, is heterozygous for the *PHGDH* c.628G>T (p.G228W) loss-of-function (LOF) variant. Consistent with our previous work modeling *PHGDH* c.628G>T (p.G228W) in CRISPR-edited control iRPE (15), donor 11 iRPE showed a decreased abundance of serine (42%) and glycine (24%) compared with non-MacTel donor control iRPE (Figure 4B, red dot). Overall, we observed that MacTel iRPE cells had a mean reduction in serine and glycine levels similar to that observed in donor plasma.

The maintenance of serine and glycine levels is critical for proper cellular function, therefore, their biosynthesis is carefully homeostatically regulated (27). We next examined whether MacTel iRPE cells were compensating for depleted pools of serine and glycine by upregulating expression levels of genes in the serine/glycine synthesis pathway. We observed a trend toward elevated expression of RNA encoding the 3 serine biosynthesis enzymes *PHGDH*, *PSAT*, and *PSPH*, whose expression increased by 27%, 31%, and 21%, respectively, in MacTel iRPE compared with control cells, but only *PSAT* expression was significantly elevated (*P* = 0.046, Figure 4C). Interestingly we found that genes encoding 1-carbon metabolism enzymes that convert serine to glycine in the cytoplasm (*SHMT1*) and mitochondria (*SHMT2*) were significantly elevated by 40% (*P* = 3 × 10^–4^) and 36% (*P* = 0.04), respectively (Figure 4C). We also found a significant 21% increase in expression of the serine membrane transporter gene *SLC1A4* (*P* = 0.02, Supplemental Figure 3B). Interestingly, the *SLC1A4* gene is located within the MacTel GWAS locus at 2p14 (18). The expression of the transcription factor *ATF4*, which can regulate serine biosynthesis, was unchanged (Supplemental Figure 3B). All donors were screened for rare coding variants in the serine/glycine biosynthesis pathway genes identified in Figure 4A (Supplemental Table 1). Apart from the previously identified PHGDH p.G228W variant in donor 11, a p.T152I PSPH variant in the unaffected control, donor 6, was also identified. The combined reduction in serine and glycine levels and the elevation of serine and glycine biosynthesis and transport genes indicated that MacTel iRPE cells had disrupted serine and glycine metabolism and thus recapitulated a major metabolic disease phenotype observed in MacTel.

### RNA-Seq identifies elevated cell stress response pathways in MacTel cells.

**Figure 5:**
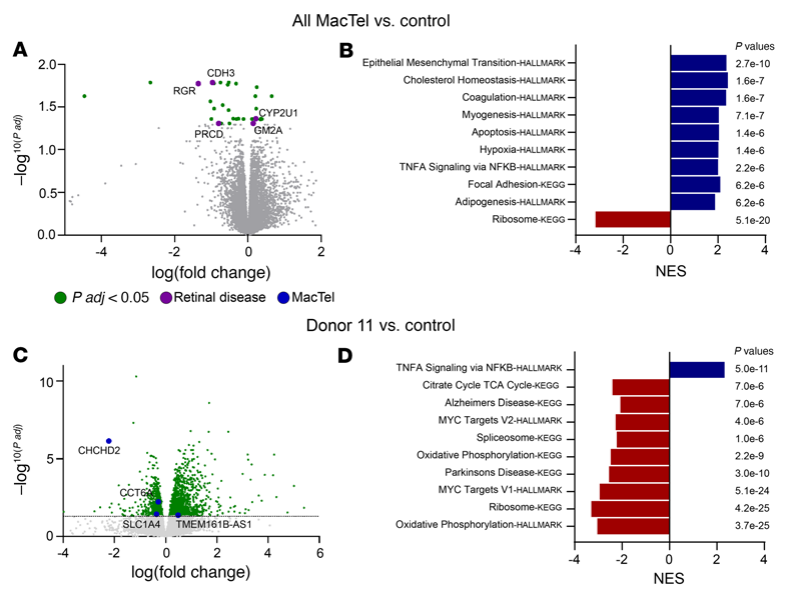
RNA-Seq pathway analysis of iRPE cells. (**A** and **C**) Enhanced volcano plot depicting all differentially expressed genes in MacTel iRPE cells compared with control iRPE cells. Colored dots indicate differentially expressed genes with an FDR of less than 0.05. Purple dots indicate retinal disease–related genes; blue dots indicate genes linked to MacTel GWAS loci; gray dots indicate genes with no significant change. p(adj), adjusted *P* value. (**B** and **D**) Graphs of the top 10 most significantly affected gene sets from GSEA with Hallmark and KEGG gene sets between differentially expressed genes comparing all MacTel iRPE (**B**) and donor 11 iRPE (**D**) cells with all control iRPE cells. Data are represented as the NES for each gene set.

To identify any potential novel defects in MacTel iRPE, we next performed RNA-Seq analysis in an untargeted screen on MacTel and control iRPE cells matured to 16 weeks. Differential gene expression between MacTel and control was determined using mixed linear models correcting for batch and related individuals. We found 31 genes with an adjusted *P* value (FDR) below 0.05 and 126 genes with an FDR below 0.1, including *SHMT1*, the most significantly upregulated gene in Figure 4C (Figure 5A and Supplemental Figure 4). We compared the significantly dysregulated genes identified in this screen (FDR <0.05) against genes linked to inherited retinal diseases and identified 5 genes: *RGR*, *PRCD*, *CDH3*, *GM2A*, and *CYP2U1* (Figure 5A). Three genes (*CDH3*, *GM2A*, and *CYP2U1*) have been directly linked to diseases with macular dysfunction. Mutations in CDH3 cause recessive juvenile macular dystrophy, a disease that shares phenotypical features with MacTel, including pigment plaques, outer retinal hyper-reflective lesions, and a loss of photoreceptors and affects a similar retinal area (28). GM2A is linked to Tay-Sachs which is a syndrome that is characterized by macular defects (29). Interestingly, 2 independent families with recessive CYP2U1 mutations leading to hereditary spastic paraplegia (HSP) also present with MacTel (30, 31). To determine whether the expression of these disease-linked genes is correlated to disease severity or phenotypic characteristics of the disease, we performed a regression analysis comparing expression of retinal disease–linked genes in donor cells with multiple clinical retinal phenotypes from the donors themselves. One donor (proband 14) showed a comorbidity of MacTel and age-related macular degeneration (AMD) and was thus excluded from the phenotypical analyses. Surprisingly, we found the strongest correlations between a depletion of macular pigment and *CDH3* (–0.87, *P* = 0.006, Spearman’s correlation coefficient *r*), and CYP2U1 (–0.85, *P* = 0.018, Spearman’s correlation coefficient *r*) expression, respectively (Supplemental Figure 5). All donors were screened for rare coding variants in these disease-linked genes (Supplemental Table 1). We identified a p.M199V variant in RGR in 2 unaffected control donors, donors 3 and 4.

We next performed GSEA using the Hallmark and Kyoto Encyclopedia of Genes and Genomes (KEGG) pathway databases to identify potential perturbations of major cellular processes and pathways in MacTel (Figure 5B and Supplemental Table 2). We identified 49 significantly enriched and 4 significantly depleted pathways in MacTel iRPE cells. Many of the enriched pathways in MacTel iRPE cells are mediators of cell stress responses including hypoxia, NF-κB, complement, ROS, as well as pathways downstream of cell stress responses including those for p53, apoptosis, and epithelial-mesenchymal transition (EMT) (Figure 5B and Supplemental Table 2). The EMT pathway, which was the most significantly enriched gene set in MacTel donor cells, is an indicator of RPE dysfunction in diseases such as AMD and proliferative vitreoretinopathy (32) (Figure 5B). RPE cells that undergo EMT lose their barrier function, allowing them to migrate into the retina, which may be observed in later stages of MacTel disease in patients whose RPE cells have been proposed to form visible pigmented plaques along aberrant vessels in the macula (33). In addition to the enrichment of cell stress response pathways, we also observed the depletion of MYC pathway targets and ribosomal proteins (Figure 5B). The reduction in ribosomal genes was consistent with a reduction in MYC signaling, as MYC primarily regulates the high energy consumption process of ribosomal biogenesis as well as central carbon metabolism, indicating a potential reduction in energy metabolism in MacTel iRPE (34, 35).

### The OXPHOS pathway is disrupted in iRPE cells from donors with early-onset MacTel.

iPSC-derived tissues are developmentally immature; they are most successfully used to model highly penetrant, early-onset diseases. In age-related diseases like MacTel, we would expect cell-intrinsic defects to be subtle, as pathologies develop with time and exposure to environmental stressors. We hypothesized that selecting MacTel donors with more severe, early-onset disease would potentially increase our ability to detect MacTel-associated differential gene expression. Therefore, we focused on iRPE cells from donor 11, a donor with early-onset MacTel with multigenerational incidences of MacTel, indicative of a more Mendelian form of the disease, with high penetrance. To ensure adequate fidelity of iRPE cells to donor 11’s genetic background, we generated 4 independently derived clones of iPSCs from donor 11. Analysis of differential expression between donor 11 and all non-MacTel donor iRPE cells identified 2,157 differentially expressed genes. In this gene set, we identified multiple differentially expressed genes that are linked to MacTel through GWAS loci and expression quantitative trait loci (eQTL) analysis (17) (Figure 5C). One gene, *CHCHD2*, located within the 7p11.2 MacTel risk locus, was the third most significantly downregulated gene in donor 11 compared with control cells (17) (log fold change [FC] = –2.2, *P* = 7 × 10^–7^) (Figure 5C). SNP genotyping of donor 11 revealed that this individual was also homozygous for the lead sentinel SNP linked to MacTel at 7p11.2 (Supplemental Table 3). The reduction of *CHCHD2* expression was consistent with the eQTL analysis, which showed a significant reduction in expression with MacTel-linked SNPs at 7p11.2 (17). CHCHD2 is a regulator of the electron transport chain and mitochondrial function (36).

A subsequent GSEA pathway analysis identified 17 enriched and 20 depleted pathways in iRPE cells from donor 11 (Figure 5D and Supplemental Table 4). Enriched pathways included the previously identified stress pathways: EMT, NF-κB, hypoxia, and complement (Figure 5D and Supplemental Tables 2 and 4). In addition to these conserved gene sets, we identified the depletion of 3 gene sets related to mitochondrial function including the hallmark OXPHOS gene set, which was the most significantly affected gene set identified in all analyses (normalized enrichment score [NES] = –3.0, *P* = 3.7 × 10^–25^) (Figure 5D). The dysregulation of these energy metabolism pathways strongly indicated a potential defect in oxidative metabolism in the iRPE cells of donor 11.

### MacTel iRPE cells have serine-independent mitochondrial dysfunction.

**Figure 6:**
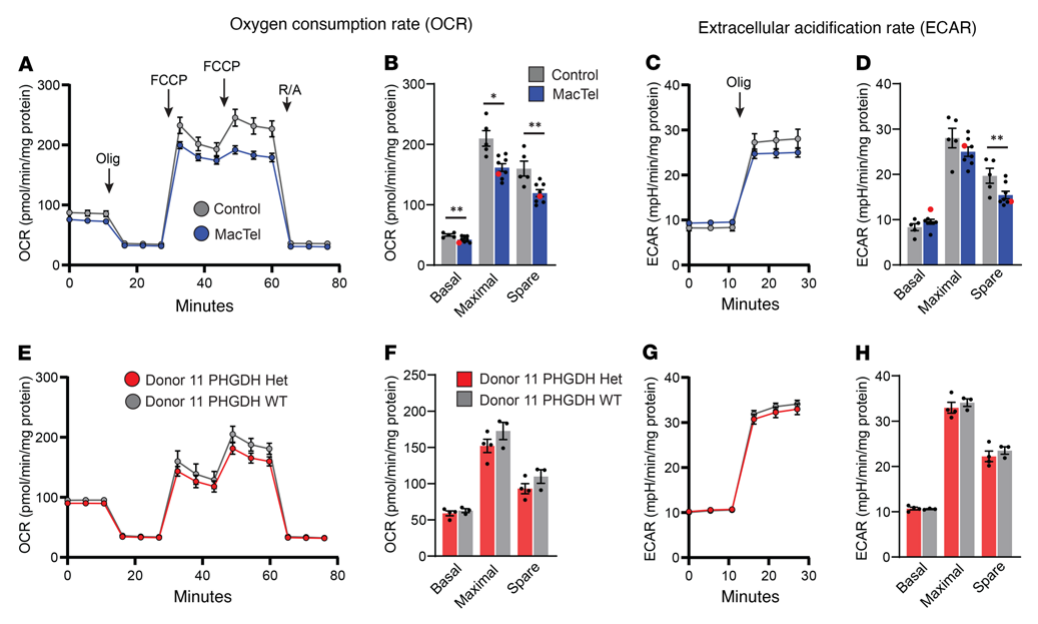
MacTel iRPE cells have reduced mitochondrial function. Bioenergetic analysis of iRPE cells measuring mitochondrial respiration (**A**, **B**, **E**, and **F**) and glycolysis (**C**, **D**, **G**, and **H**). (**A**–**D**) Comparison between control (*n* = 5 donors) and MacTel (*n* = 8 donors) iRPE cells. (**E**–**H**) Comparison between donor 11 iRPE cells (Donor 11 PHGDH Het, *n* = 4 clones) and donor 11 iRPE cells with the PHGDH G228W variant CRISPR corrected to WT (Donor 11 PHGDH WT, *n* = 3 clones). (**A** and **E**) OCR measurement traces. (**B** and **F**) Basal OCR represents the time point prior to Olig treatment, maximal OCR represents the time point following the second FCCP treatment, and spare OCR represents the difference between the basal and maximal time points. (**C** and **G**) ECAR measurement traces. (**D** and **H**) Basal ECAR represents the time point prior to Olig treatment, maximal ECAR represents the third time point following Olig treatment, and spare ECAR represents the difference between the basal and maximal time points. (**B** and **D**) Donor 11 is represented by a red dot. (**A**–**D**) Data are represented as the mean of the donors ± SEM. (**A**–**D**) Each individual donor represents the average of at least 2 independent clones, each run in 15–22 technical replicates. *P < 0.05 and **P < 0.01, by mixed linear modeling. (**E**–**H**) Data are represented as the mean of the clones ± SEM. Each individual clone is the average of 12–16 technical replicates. Statistical significance was determined by 2-tailed Student’s *t* test.

Given the depletion of gene sets linked to energy metabolism, we next sought to determine whether mitochondrial function is disrupted in MacTel iRPE cells, including those from donor 11, by measuring the cellular oxygen consumption rate (OCR) in all donor iRPE cells using the mitochondrial stress test with a Seahorse Analyzer (Figure 6, A and B). Surprisingly, we observed a significant reduction in the basal OCR (15%, *P* = 0.003), maximal OCR (23%, *P* = 0.02), and spare capacity (25%, *P* = 0.03) in MacTel iRPE cells (Figure 6, A and B). These data indicate a broad reduction in mitochondrial function in MacTel iRPE cells, characterized by both lower resting respiration and reduced maximal capacity. The reduced mitochondrial respiration in MacTel donors was also conserved within family groups, in which MacTel donor cells had lower OCR rates than did donor cells from control family members (Supplemental Figure 6, A and B). We next measured mitochondrial genome content in MacTel and control iRPE cells and found no difference in the number of mitochondria between the 2 groups of cells (Supplemental Figure 6C). When we measured the extracellular acidification rate (ECAR), an indicator of glycolytic function, we observed a significant decrease in the spare ECAR capacity (~21%, *P* = 0.004) in MacTel iRPE cells (Figure 6, C and D). The dysregulation of central metabolic pathways and the reduced metabolic function indicated a shared cell-intrinsic energy metabolism defect in MacTel donor cells.

In various cell models, a reduction in serine biosynthesis has been shown to disrupt mitochondrial function directly (37) and indirectly through accumulation in 1-dSLs (38, 39). We next determined whether the observed energetic defects are linked to either reduced serine biosynthesis or accumulation in 1-dSLs. First, we tested mitochondrial function in control iRPE cells under serine and glycine deprivation for 1 week, a period that elevates 1-dSL concentrations in iRPE 6-fold (15), as well as in media with 1 μM 1-deoxysphinganine, a concentration we have shown to be toxic (14). Using the Seahorse Analyzer mitochondrial stress test, we observed no reduction in mitochondrial or glycolytic capacity under either condition (Supplemental Figure 6, D and E). Reduced cellular energetics could also be linked directly to reduced PHGDH function. In cancer cells, knockdown of PHGDH that causes reduced serine synthesis also reduces central carbon metabolism (37). Contrary to this, we have previously shown that the MacTel-associated LOF PHGDH G228W variant carried by donor 11 causes a significant decrease in serine synthesis and abundance in iRPE without altering oxygen consumption or glycolysis (15). The patient with early-onset MacTel, donor 11, has a distinct defect in OXPHOS as shown by RNA-Seq (Figure 5D) and mitochondrial stress testing (Figure 6B, red dots). This donor is also heterozygous for the MacTel-associated LOF PHGDH G228W variant (15). In this case, PHGDH LOF could be contributing to energetic defects in this particular genetic background. To test this, we generated multiple independent clones of donor 11 iRPE cells with the PHGDH G228W variant corrected to WT using CRISPR editing (Supplemental Figure 7A). We validated that editing the PHGDH G228W variant back to WT in donor 11 iRPE cells rescued PHGDH function by quantifying isotope enrichment of serine in the presence of [U-^13^C_6_] glucose and found that serine labeling and abundance were increased in the CRISPR-corrected PHGDH WT iRPE cells compared with unmodified donor 11 iRPE cells (Supplemental Figure 7B). When we tested mitochondrial function in these cells, we found no significant increase in mitochondrial respiration or glycolytic capacity in donor 11 iRPE cells homozygous for WT *PHGDH* compared with donor 11 iRPE cells heterozygous for PHGDH (Figure 6, E–H). Finally we sequenced the transcriptome of these cells and did not observe any significant changes in gene expression, including that of genes reported in Figure 5C (Supplemental Figure 7, C and D). These experiments strongly suggest that the observed mitochondrial dysfunction in MacTel donor iRPE cells was independent of decreased serine and glycine abundance, defects in serine biosynthesis, or accumulation of 1-dSLs.

## Discussion

Through the analysis of iPSC-derived RPE cells from patients with or without macular degenerative disease, we document transcriptional and metabolic abnormalities that revealed what we believe to be a previously undescribed phenotypic change in MacTel tissues. We leveraged our donor-derived iRPE library to investigate, for the first time to our knowledge, pathological phenotypes in iRPE as a model for MacTel, a retinal metabolic disorder. We provide a comprehensive multi-omics analysis of MacTel iRPE cells to validate that they exhibit disrupted serine synthesis, a cellular pathology linked to MacTel, and elevated cell stress. We also describe reduced mitochondrial function as a new disease-associated metabolic phenotype independent of serine synthesis. We suggest that using such multi-omics analyses of iPSC-derived tissues from diseased and unaffected individuals may be useful to uncover pathological mechanisms of other complex genetic diseases.

This study provides the first evidence, to our knowledge, that defects in energy metabolism could be a contributing pathological feature in MacTel. RNA-Seq pathway analysis of a donor with early-onset MacTel, donor 11, identified a standout deficit in genes involved in OXPHOS. A subsequent bioenergetic analysis with a mitochondrial stress test in all MacTel and control iRPE cells identified a broad reduction of mitochondrial function as well as a reduction in glycolytic capacity in MacTel donor iRPE cells, indicating a shared dysfunction in energy metabolism. Furthermore, we show that this disease-linked phenotype is independent from previously described serine-linked mechanisms of disease. Mitochondrial defects are associated with multiple retinal disorders, as the retina is particularly sensitive to mitochondrial dysfunction due to its high energy demand and elevated oxidative stress (40–43). The role of mitochondrial dysfunctions in pathological mechanisms can be due to their contribution to energy production, the folate cycle, oxidative stress, biosynthesis of iron-sulfur clusters, calcium homeostasis, or regulation of apoptosis (44, 45), and they can be caused by mutations in any of the 37 genes in the mitochondrial genome or the 1,000–2,000 estimated secondary mitochondrial genes in the nuclear genome.

*Do mitochondrial defects contribute to MacTel pathology?* Histological characterizations in MacTel patient retinas and the discovery of multiple comorbidities of MacTel with mitochondrial diseases suggest that mitochondrial dysfunction could play a role in the etiology of MacTel. In postmortem samples of MacTel donor retinas, structural mitochondrial defects have been detected as early indicators of cellular dysfunction in the macular Müller glia (46). Recent clinical studies have also reported the diagnosis of MacTel in multiple individuals with severe LOF mutations either in genes of the mitochondrial genome or in genes that directly impact mitochondrial function (30, 31, 42). In 2 independent cases, patients who were initially referred with the mitochondrial disease chronic progressive external ophthalmoplegia also showed retinal characteristics similar to those of patients with MacTel (42). Genomic testing in these patients identified large mitochondrial genomic deletions (3–5 kb) spanning ND4 and ND5, suggesting a potential link between primary mitochondrial defects and MacTel. Secondary mitochondrial mutations in the nuclear genome that cause mitochondrial dysfunction have also been linked to MacTel, in which multiple individuals diagnosed with HSP caused by separate variants in CYP2U1 have been found to have early-onset forms of MacTel (30, 31). CYP2U1 is localized to the mitochondria, and donor-derived tissue with CYP2U1 mutations have reduced mitochondrial function, with decreased basal and maximal OCRs and increased mitochondrial fragmentation (47, 48). Recent analysis of *CYP2U1*-knockout mice and tissue from patients with HSP indicates that CYP2U1 hydrolyzes vitamin B2, regulating the folate pathway, where LOF directly inhibits the electron transport chain (ETC) (49). It is also interesting to note that *CYP2U1* was one of the few significantly upregulated genes in MacTel iRPE cells, as identified by RNA-Seq, and its expression correlated with disease severity in the retinas of donors. Increased CYP2U1 expression in MacTel iRPE cells could indicate an elevated demand for cellular functions dependent on CYP2U1, such as folate synthesis. The correlation of reduced CYP2U1 expression with disease severity in patients may suggest that cells less able to compensate for LOF with elevated CYP2U1 expression result in poorer outcomes for patients. Although it is currently unclear what role reduced mitochondrial function may play in MacTel pathology, the decrease in mitochondrial function we observed here, as well as the link between the presence of multiple different mitochondrial gene variants and MacTel, strongly suggests that mitochondrial dysfunction can contribute to the onset of MacTel.

*What causes reduced energy metabolism in MacTel?* Whole-exome sequencing (WES) and GWAS analyses of MacTel populations have not yet identified any variants or nuclear genomic loci that are clearly associated with mitochondrial function, and mitochondrial GWAS analysis has not yet, to our knowledge, been performed. However, it is important to note that the function of the majority of MacTel-associated GWAS loci have not been elucidated and may include noncoding variants that influence the regulation of energy metabolism. One potential candidate is the *CHCHD2* gene, which lies within the MacTel-linked GWAS peak at the 7p11.2 locus (17). In this study, we found that *CHCHD2* was one of the most significantly downregulated genes in donor 11, who is also homozygous for the MacTel-associated sentinel allele at 7p11.2. In an eQTL analysis by Bonelli et al. (17) revealed that reduced expression of *CHCHD2* is significantly associated with the MacTel-associated locus at 7p11.2 and is the most affected gene at that locus. CHCHD2 is a regulator of mitochondrial function, where knockout models show reduced basal and maximal OCRs, similar to what we report here in MacTel iRPE cells (36, 50). This suggests a potential causal link between reduced *CHCHD2* expression and the mitochondrial defects observed in donor 11. Future work identifying functional variants in the 7p11.2 locus will be critical to understanding the disease mechanism.

Through depletion of serine and glycine in culture media, the addition of exogenous 1-deoxysphinganine and CRISPR correction of a LOF PHGDH in donor 11, we definitively show that the observed mitochondrial defects were independent of serine or 1-dSLs in iRPE cells. Conversely, it is unlikely that reduced mitochondrial function contributed to the reduced serine and glycine levels observed, as pharmacological inhibition of the ETC in human RPE does not alter serine abundance (51). Furthermore, mouse models and patients with severe mitochondrial dysfunction have elevated levels of serine, glycine, and alanine (52, 53). The pathological consequences of both altered serine metabolism and OXPHOS may converge on a downstream cellular process, or both may be the result of an independent upstream defect.

### Elevated cell stress in MacTel iRPE.

Using RNA-Seq pathway analysis, we discovered the enrichment of multiple cell stress pathways in MacTel iRPE cells. The enrichment of these pathways suggests that MacTel iRPE cells were in a disease state, however, it was difficult to determine whether these specific pathways indicated a generic cell stress response to a disease state or highlighted a MacTel-specific disease mechanism. Dysregulation of many of these pathways is consistent with the RPE dysfunction observed in other retinal diseases. The most significantly enriched pathway, the EMT pathway, is an important marker of RPE dysfunction in AMD and proliferative vitreoretinopathy (32). EMT induction drives RPE dedifferentiation, leading to increased cell migration and proliferation, therefore, its enrichment is consistent with the downregulation of CDH3, a cadherin required for RPE tight junctions (54), in MacTel iRPE cells. This would suggest that the observed correlation of reduced CDH3 expression with disease severity in patients with MacTel may indicate the ability of patients’ RPE to remain in a healthy epithelial state. The elevation of hypoxia and ROS in RPE, whose pathways are enriched in MacTel iRPE, is also linked to AMD and has been shown to alter lipid metabolism as well as glycolysis in RPE, leading to photoreceptor degeneration (55, 56). Dysregulation of the cholesterol and complement pathways in RPE, also enriched in MacTel iRPE, is central to the development of AMD (57). Furthermore, dysregulation of each of these pathways is linked to mitochondrial stress. In this model, it is possible that chronic cell stress could result in repression of mitochondrial function. Conversely, reduced mitochondrial function could also lead to an increase in cell stress signaling. MacTel is a genetically complex disease, therefore, the cause of reduced mitochondrial function in MacTel iRPE cells is likely heterogeneous, making it difficult to pinpoint the exact causative mechanism(s) of reduced mitochondrial function in all iRPE cells. However, the identification of mitochondrial dysfunction and multiple cell stress pathways as convergent phenotypes in MacTel RPE provides a better understanding of the molecular complexity of the MacTel disease state.

### The role of the RPE in MacTel.

Postmortem analyses of retinas from patients with MacTel have previously provided strong evidence that the RPE contributes to the pathology observed in MacTel (20). RPE dysfunction has also been shown to be central in other diseases of the macula such as AMD (56, 57). Although it is unknown why MacTel is restricted to the macula, the region’s specialized function and anatomy may impart unique strains on the photoreceptors and Müller glia of the macula, making them more susceptible to defects in the support role of RPE. The macula has a higher concentration of photoreceptors compared with the periphery, leading to increased oxidative stress, and macular Müller glia have elevated serine synthesis and glycolysis compared with the periphery, indicating unique metabolic demands (58). Our data do not exclude the possibility that other cell types in the retina contribute to MacTel as well, and because of their overlapping functions with the RPE, determining whether Müller glia have similar defects will be critical to better understand the disease mechanism. Furthermore, our data do not account for the cumulative effects of aging and environmental stress. Since clinical MacTel metabolic phenotypes are observed systemically in patients, it is likely that systemic metabolic stressors also contribute to MacTel (13, 14). The lack of external stressors and aging may account for our inability to detect phagocytosis defects in MacTel iRPE, despite the fact that RPE phagocytosis defects have been observed in the retinas of patients with MacTel (20). However, our data suggest that reduced serine synthesis and mitochondrial function in the RPE are primary defects that may contribute to catastrophic defects of RPE function following the cumulative damage from aging and repeated stress over a lifetime, both of which contribute to the onset of MacTel.

Through the analysis of donor-derived iRPE, we have provided transcriptional and metabolic insights that uncover what we believe to be a previously undescribed phenotypic change in MacTel tissues. This approach should be useful in the analysis of other poorly understood non-Mendelian diseases with an incompletely characterized genetic component. With regard to MacTel, these discoveries will guide future efforts to better understand this complex disease and direct the development of personalized treatment.

### Limitations of the study.

In this study, we present a comparison of donor-derived iRPE through functional cellular assays, transcriptomics, and metabolic profiling and observed what to our knowledge is a previously undescribed phenotypic change in the cells of patients with MacTel. Although we demonstrate a common disease-linked cellular defect in patient-derived iRPE, future studies that expand disease profiling in other cell types linked to MacTel, such as photoreceptors and Müller glia, will be helpful to better understand the role of the RPE in the pathological mechanism of MacTel. Together with the experiments presented here, these efforts will help standardize the use of patient-derived cell models as a powerful system for studies of human disease.

## Methods

### WES analysis.

All samples were sequenced either at the Institute of Genomic Medicine (IGM), Columbia University, with the IDT’s Exome Research Panel, version 1 (differing capture kits), or off-site at Macrogen/Psomagen using Agilent V5 or V7 kits. Regardless of the sequencing facility, all raw data were processed using the IGM alignment and annotation pipeline for standardized analysis outcomes. Samples were sequenced on the Illumina next-generation sequencing machines using the DRAGEN Bio-IT Platform, version 2.5.1 to align reads to the Genome Reference Consortium’s Human Build 37, calling variants in accordance with the Genome Analysis Tool Kit (GATK, version 4.0.2.1) Best Practices Workflow, using ATAV (version 7.0.16), an IGM variant-calling pipeline.

### Generation and maintenance of iPSCs.

The human (hiPSC) lines were derived from PBMCs obtained from donors. Reprogramming was performed by the Harvard and Salk iPS core facilities using the Sendai virus for reprogramming factor delivery. All cell lines used were verified for a normal karyotype (Cell Line Genetics) and contamination free. hiPSCs were maintained on Matrigel-coated (BD Biosciences) plates with mTeSR1 medium (STEMCELL Technologies). Cells were passaged every 3–4 days at approximately 80% confluence. Colonies containing clearly visible differentiated cells were marked and mechanically removed before passaging.

### Differentiation of RPE cells.

For directed differentiation of RPE cells, 10 mM nicotinamide (MilliporeSigma) and 31 ng/mL activin A–based (Peprotech) protocols were used according to those previously described (59). Confluent iPSC cultures were switched to differentiation media containing Knockout DMEM (Thermo Fisher Scientific, 10829018), 20% Knockout Serum Replacement (Thermo Fisher Scientific, 10828028), 2 mM l-glutamine (Thermo Fisher Scientific, 25030081), 1% penicillin/streptomycin (Thermo Fisher Scientific, 15140-122), 10 mM nicotinamide (MilliporeSigma, N0636), 0.1 mM β-mercaptoethanol (Thermo Fisher Scientific, 21985-023), and 1% nonessential amino acid (NEAA) solution (Thermo Fisher Scientific, 11140). Differentiating iPSCs were maintained in differentiation media for 3 weeks, then switched to differentiation media plus 31 ng/mL activin A (PeproTech, 120-14E) for 2 weeks, and then switched back to differentiation media. Following differentiation, pigmented colonies were manually excised and plated on growth factor–reduced Matrigel (Corning, 356230) for expansion. RPE cells were passaged once at 1 to 6 for expansion before being passaged and plated for assay conditions. For experiments on Transwell inserts, we used 0.4 μM pore size inserts coated with fibronectin (Corning, 47743-654). RPE cells were plated in assay conditions at 50% confluence. In each assay, RPE cells were plated at the same time and matured for a minimum of 16 weeks past the point of confluence before being assayed. Following iPSC derivation, validation of clonal quality and RPE cell differentiation and of iRPE cell functional maturity was done using 2 or more mature iRPE cells from independent iPSC clones for each donor.

### Cell lines.

Human fetal RPE cells (Lonza, 00194987) were cultured according to the product protocol using the RtEGM growth medium kit (Lonza, 00195409). Cells were not expanded beyond passage 4. For assays, cells were matured for 2 weeks at confluence and then harvested. The cells were derived from a male donor.

### Mesoscale.

Cells were incubated with fresh media at 37°C for 24 hours. Media samples were collected from apical and basal chambers and diluted 5 times in dilution buffer. VEGF and PEDF levels were determined using a Mesoscale Discovery (MSD) MULTI-SPOT immunoassay following the manufacturer’s protocol. Samples were added to each well of an MSD plate that was precoated with capture antibodies on a specific spot. Plates were incubated and shaken for 2 hours at 300 rpm at room temperature (RT). Plates were then washed 3 times with wash buffer, then 25 μL detection antibodies conjugated with electrochemiluminescent labels (SULFO-TAG) were added. Plates were shaken at RT for 2 hours and then washed 3 times with wash buffer. After the addition of read buffer, plates were read using the MSD SECTOR Imager. Absolute protein concentrations were determined using a standard curve.

### RNA isolation and quantitative RT-PCR.

Total RNA was purified from frozen tissues using TRIzol Reagent (Life Technologies, Thermo Fisher Scientific) according to the manufacturer’s instructions. First-strand cDNA was synthesized from 400 ng total RNA using the High-Capacity Reverse Transcriptase kit (Applied Biosystems) according to the manufacturer’s instructions. Individual 10 μL SYBR Green Master Mix (Applied Biosystems) real-time PCR reactions consisted of 2 μL diluted cDNA, 5 μL Power Up SYBR Green (Applied Biosystems), and 1 μL of each of the 5 μM forward and reverse primers. The PCR was carried out on 384-well plates on a QuantStudio Real-Time PCR system (Applied Biosystems) using a 3-stage program: 95°C for 10 minutes, 40 cycles of 95°C for 20 seconds, 60°C for 20 seconds, and 72°C for 20 seconds. PCR data for intergene comparison were corrected for primer efficiency. Samples were normalized to the internal loading control 36B4. The primers and primer sequences are provided in Supplemental Table 5.

### Phagocytosis assay.

Photoreceptor outer segments (POSs) were isolated from fresh porcine eyes (Sierra for Medical Science, http://www.sierra-medical.com) as previously described (60) and conjugated with Alexa Fluor 405 NHS Ester (Invitrogen, Thermo Fisher Scientific, A30100) as described in the product manual. fPOS preparations were quantified using Bradford assays. fPOS aliquots were stored at –80°C. Cells were treated with 10 μg/cm^2^ fPOS and incubated at 37°C for 5 hours. All media were removed, and cells were rinsed with Dulbecco’s PBS (–/–) and then treated with trypsin for 5 minutes at 37°C. Cells were dissociated in trypsin and then diluted 1:1 in FACS buffer (1× PBS [–/–], 2.5 mM EDTA, 25 mM HEPES, pH 7.0, 1% heat-inactivated FBS, 1:2,000 DRAQ5). Samples were immediately analyzed on a ZE5 YETI (Bio-Rad). Cells were gated according to DRAQ5 labeling, and 40,000 events were collected per sample. Data were processed using FlowJo software (BD Biosciences).

### Cell culture protocol for metabolite abundance measurements.

To measure metabolite levels in RPE cocultured media, iRPE cells were switched to serine- and glycine-depleted custom RPE media substituted with custom Knock Out DMEM media and custom knock out serum replacement (KSR) (outlined below). iRPE cells were switched to custom media with 25 mM glucose and depleted of serine and glycine for 24 hours, allowing for acclimation to biosynthetic requirements. After 24 hours, cells were switched to fresh depleted media containing 25 mM glucose (for standard gas chromatography/mass spectrometry [GC/MS] metabolite abundance and sphingolipid abundance) and cultured for 24 hours. Twenty-four hours later, cocultured media were removed, and samples were spun down and snap frozen. Metabolite measurements from GC/MS were normalized to the protein content of the RPE culture using a bicinchoninic acid (BCA) protein assay (Pierce, Thermo Fisher Scientific). Isotope tracing using [U-^13^C_6_] glucose was performed as previously described (15).

### Custom depleted RPE media.

Custom Knock Out DMEM without glucose, pyruvate, serine, or glycine was manufactured by Gibco (Thermo Fisher Scientific), with all additional proprietary components unchanged. Custom KSR formulation was provided as previously described (15).

### RNA-Seq.

For each sample, total RNA samples (100 ng) were prepared into sequencing libraries using NEB Ultra II RNA-Seq library prep kits with polyA selection and 12 PCR cycles following the manufacturer’s recommended protocol. Libraries were dual indexed and loaded onto an Illumina NextSeq 2000 targeting 20 M 100 base reads per sample. Raw reads are available in the NCBI’s BioProject database (accession no. PRJNA937864). Reads were mapped to the human genome build Hg38 using the STAR aligner (61), suppressing multimapping reads. Gene counts were calculated using the Rsubread featureCounts (62) module, and those with at least 1 count per million in at least 3 samples were retained for further analysis. Using the limma (63) and edgeR (64) packages, trimmed mean of M (TMM) normalization was applied across samples and genes differentially expressed between MacTel and control donors were calculated using voom (65) and eBayes modules, correcting for sequencing batch and with a random effect for related donors. Donor 11 was compared with controls in a similar manner.

Hallmark and KEGG GSEA was performed using the limma fry module and the fgsea package (66).

### Retinal image analysis, grading, and correlation with gene expression markers.

Retinal imaging markers known to be associated with disease severity (67–70) as well as phenotypical characteristics of the disease (8) were evaluated on multimodal retinal images, and both eyes of each participant were considered.

On the basis of color fundus photographs and fluorescein angiographic findings, eyes were divided into disease stages 1–5 according to the classification proposed by Gass and Blodi (69). OCT volume scans were analyzed for the presence of ellipsoid zone (EZ) loss, outer retinal hyper-reflective lesions, and neovascular membranes as previously described (67, 68).

For quantification of EZ loss, OCT volume scans (97 B-scans, 15° horizontal × 10° vertical; Spectralis, Heidelberg Engineering) were analyzed using the 3D view panel as previously described (69).

Macular pigment optical density (MPOD) maps were analyzed, and eyes were graded into MPOD distribution classes 1–3 using the classification system proposed by Zeimer et al. (71) Briefly, the latter differentiates between 3 depletion patterns of macular pigment (“MPOD-classes”) describing a progressive loss of pigment with increasing pigment classes.

For correlation analyses, parameters from fellow eyes were collapsed into a summary value for each donor, and Pearson and Spearman correlation coefficients, respectively, were used to describe associations between gene expression markers and retinal morphologic alterations.

Gene expression of CYP2U1, CDH3, PRCD, GM2A, and RGR in iRPE cells were measured by quantitative PCR (qPCR).

### GC/MS analysis.

Polar metabolites from media were extracted using a methanol/water (8:1) solution with inclusion of norvaline as an internal standard. Samples were vortexed for 10 minutes and centrifuged at 16,000*g* for 10 minutes. The upper aqueous layer was collected and dried under vacuum, and then 2% (w/v) methoxyamine hydrochloride (Thermo Fisher Scientific) in pyridine was added and incubated at 37°C for 60 minutes. Samples were then silylated with *N*-tertbutyldimethylsilyl-*N*-methyltrifluoroacetamide (MTBSTFA) with 1% *tert*-butyldimethylchlorosilane (tBDMS) (Regis Technologies) at 37°C for 30 minutes. The derivatized metabolites were analyzed by an Agilent 7890B gas chromatograph equipped with a DB-35MS column (30 m [length] × 0.25 mm [inner diameter] J&W Scientific, Agilent) and connected with an Agilent 5977C mass spectrometer. For metabolite separation, the gas chromatograph oven was held at 100°C for 2 minutes, followed by an increase in the temperature to 300°C at a ramp rate of 10°C/min, holding for 3 minutes. Mass spectrometric scanning was performed over an *m/z* range of 100–650. The quadrupole was held at 150°C. Metabolite abundances were calculated by normalizing metabolite ion counts to the norvaline internal standard, and protein abundance was determined by BCA protein assay (Pierce, Thermo Fisher Scientific).

### Extracellular metabolic flux analysis.

The OCR and ECAR were measured on an Agilent Seahorse XFe96 Analyzer (Seahorse Biosciences) following the manufacturer’s instructions. Cells were plated 24 hours prior to analysis at a density of 5 × 10^4^ cells per well on a Seahorse XF96 V3 PS cell culture microplate (catalog 101085) coated with Synthemax (Corning, catalog CLS3535). RPE culture media were changed to Custom Knock Out DMEM and incubated for 1 hour at 37°C without a CO_2_ supply prior to the assay. Custom Knock Out DMEM (Gibco, Thermo Fisher Scientific) for the assay was manufactured without phenol red, glucose, serine, glycine, or sodium bicarbonate and was supplemented with 12 mM glucose, 10 mM HEPES, 5 mM sodium pyruvate, and 2 mM l-glutamine at a pH of 7.4. Assay parameters were set at a 3-minute mix and a 2-minute measurement in 4 steps: (a) 2 μM oligomycin (Olig) (MilliporeSigma); (b) 0.5 μM carbonyl cyanide-4 (trifluoromethoxy) phenylhydrazone (FCCP) (MilliporeSigma); (c) repeat injection of 0.5 μM FCCP to ensure that the maximal OCR was achieved; and (d) 1 μM rotenone (MilliporeSigma) and 1μM antimycin (MilliporeSigma). Following the OCR and ECAR assay, cells were quantified using the CyQUANT Cell Proliferation Assay (catalog C7026). Each OCRand ECAR measurement was normalized to the corresponding cell quantity in that well. Each clone of WT and *PHGDH* p.Gly228Trp iRPE cells was run in 22 separate wells and averaged. Wells with defective readings were removed.

### Mitochondrial genome counts.

Total DNA was extracted using the QIAGEN DNeasy Blood and Tissue Kit (catalog 69504), and mitochondrial DNA content was determined by measuring the mitochondrial gene *CoxII* (Mt-Co_2_) (forward primer, CCTGCGACTCCTTGACGTTG; reverse primer, AGCGGTGAAAGTGGTTTGGTT), normalized to genomic DNA Rip140 (*Nrip1*) (forward primer, TGATGCCTCTAT TTTCCCAA; reverse primer, ATCCCCTCCACCCAATTTTT) via qPCR.

### CRISPR genome editing.

To generate the c. 682T>G substitution in PHGDH, we designed a guide RNA (gRNA) for a targeted double-stranded break and a single-stranded DNA oligonucleotide (ssODN) with the desired substitution for homologous recombination. We generated 3 independent homozygous WT clones. Four independent iPSC clones were selected from iPSCs that underwent the same protocol without obtaining a 682T>G substitution. The gRNA was designed using Benchling (https://www.benchling.com/crispr) and cloned it into the px330-puro-eGFP plasmid (gifted by Joseph Gleeson, UCSD, La Jolla, California, USA) using the FastDigest BpiI restriction enzyme (Thermo Fisher Scientific). The ssODN was designed with the 682T>G substitution and substitutions at the gRNA PAM site that disrupted the PAM site without altering the amino acid sequence of PHGDH: gRNA, TGGAGGGATCGTGGACGAA; ssODN, CCCCAGCAGGAAGATGCTTCGCTTTCTTCCAGGCTTGCTGAATGACAACACCTTTGCCCAGTGCAAGAAGGGGGTGCGTGTGGTGAACTGTGCACGGGGAGGGATCGTGGACGAAGGCGCCCTGCTCCGGGCCCTGCAGTCTGGCCAGTGTGCCGGGGCTGCACTGGACGTGTTTACGGAAG.

We transfected the control iPSC line with the gRNA containing plasmid (1 μg) and ssODNs (10 μM) using the Neon Transfection System (Thermo Fisher Scientific) with two 30-second 850 V pulses. Transfected iPSCs were selected using puromycin selection. Clones were established from single-cell plating of puro-selected iPSCs and manually excised from individual iPSC clusters following 10 days of expansion. Sequences were validated using Sanger sequencing and the following primers: forward, 5′-TGGCCCAGATCCATAACAGG-3′; reverse, 5′-TGTAAAACGCCAGGCTTTCC-3′.

### Transmission EM.

Cells grown to confluence in Transwell culture plates were fixed in oxygenated 2.5% glutaraldehyde (72) in 0.1 M sodium cacodylate buffer (pH 7.3) for 15 minutes at 37°C and then kept at 4°C overnight. After washing in 0.1 M sodium cacodylate buffer, the samples were postfixed in buffered 1% osmium tetroxide for 1 hour at RT, rinsed in the same buffer, and then treated with 0.5% tannic acid in 0.05 M sodium cacodylate buffer for 30 minutes in the dark followed by a wash in buffered 1% Na_2_SO_4_ in the dark. Samples were washed once in buffer followed by several washes in ddH_2_O. Samples were dehydrated through a graded series of ethanol followed by 2-hydroxypropyl methacrylate (HPMA) and infiltration in LX-112 (Ladd) epoxy resin and polymerization at 60°C. Ultrathin sections (silver) were cut using a Leica UC6 ultramicrotome and poststained with aqueous uranyl acetate and lead citrate. Sections were imaged at 80 kV with a Thermo Fisher Scientific FEI Morgagni 268 transmission electron microscope, and images were acquired with a Tietz 2k CMOS camera.

### Statistics.

Data from qPCR, metabolite abundance, and metabolic flux experiments were log transformed, and differences between MacTel and control donor cells identified using mixed linear models in the R lmerTest package, with fixed effects for batch and random effects for related donors. Tidymodels and Tidyverse suites and GraphPad Prism (GraphPad Software) were used to compare statistical results as well as for data transformation and plotting. Data from CRISPR-corrected iRPE cells in Figure 6 were analyzed using a 2-tailed Students *t* test.

### Study approval.

Participants were recruited from the MacTel Natural History and Observation Registry Study (NHOR) from a single-center cohort at the Moran Eye Center of the University of Utah (Salt Lake City, Utah, USA). Protocol details of the study have been published previously (24). The diagnosis of MacTel was based on fundoscopy, spectral domain optical coherence tomography (SD-OCT), MPOD, and fluorescein angiography (FFA) (8) and was confirmed by the Moorfields Eye Hospital Reading Center. Controls were age- and sex-related healthy probands without any evidence of retinal diseases and clear media.

Patients and controls underwent a full clinical examination, including best-corrected visual acuity (BCVA) testing, dilated fundoscopy, color fundus photography (Topcon), SD-OCT (volume scans of 15 mm × 10 mm [high-resolution mode, 97 scans], Spectralis, Heidelberg Engineering, Heidelberg, Germany), infrared imaging, blue-light reflectance imaging, and fundus autofluorescence (all scanning laser ophthalmoscopy [SLO], Heidelberg Engineering). MPOD was measured as previously described using a modified 2-wavelength Heidelberg Retina Angiograph (Heidelberg Engineering) (73, 74). Participants then had fluorescence lifetime imaging ophthalmoscopy (FLIO) of their maculas on a prototype instrument supplied by Heidelberg Engineering. Additionally, fluorescein angiography (SLO, Heidelberg Engineering) was performed only in patients with MacTel.

### Study approval.

The study was approved by the local ethics committee of the University of Utah and was conducted in accordance with the declaration of Helsinki. All participants provided written informed consent.

## Author contributions

KTE, SG, RF, SHP, ST, EAM, MW, MEDR, AJ, CM, and MLG designed and performed experiments. BREA performed statistical analysis and analyzed the RNA-Seq data. LS, BH, and PSB examined patients and collected blood samples. TN and RA analyzed WES. BREA, SF, and MB assisted with GWAS analysis. Study design was conceptualized by KTE and MF. KTE wrote the manuscript with input from all co-authors.

## Supplementary Material

Supplemental data

## Figures and Tables

**Table 1:**
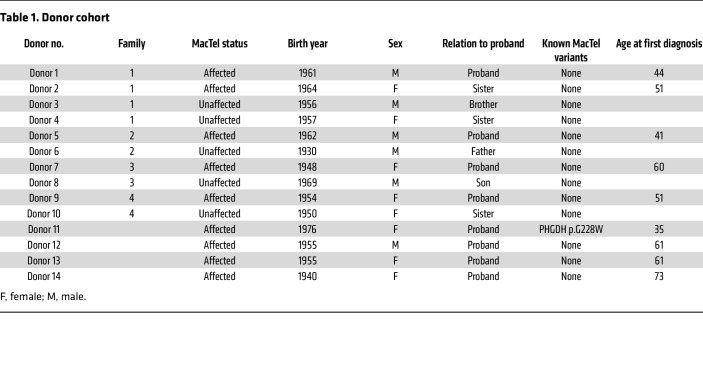
Donor cohort

## Version 1 05/01/2023

Electronic publication

## Version 2 05/03/2023

Updated author name
