# Supplementary material for: iPSC–derived retinal pigmented epithelial cells from patients with macular telangiectasia show decreased mitochondrial function
Source: J Clin Invest. 2023 May 1;133(9):e163771. doi: 10.1172/JCI163771 (PMC10145939; doi:10.1172/JCI163771)
Supplement: Supplemental data [file jci-133-163771-s008.pdf]

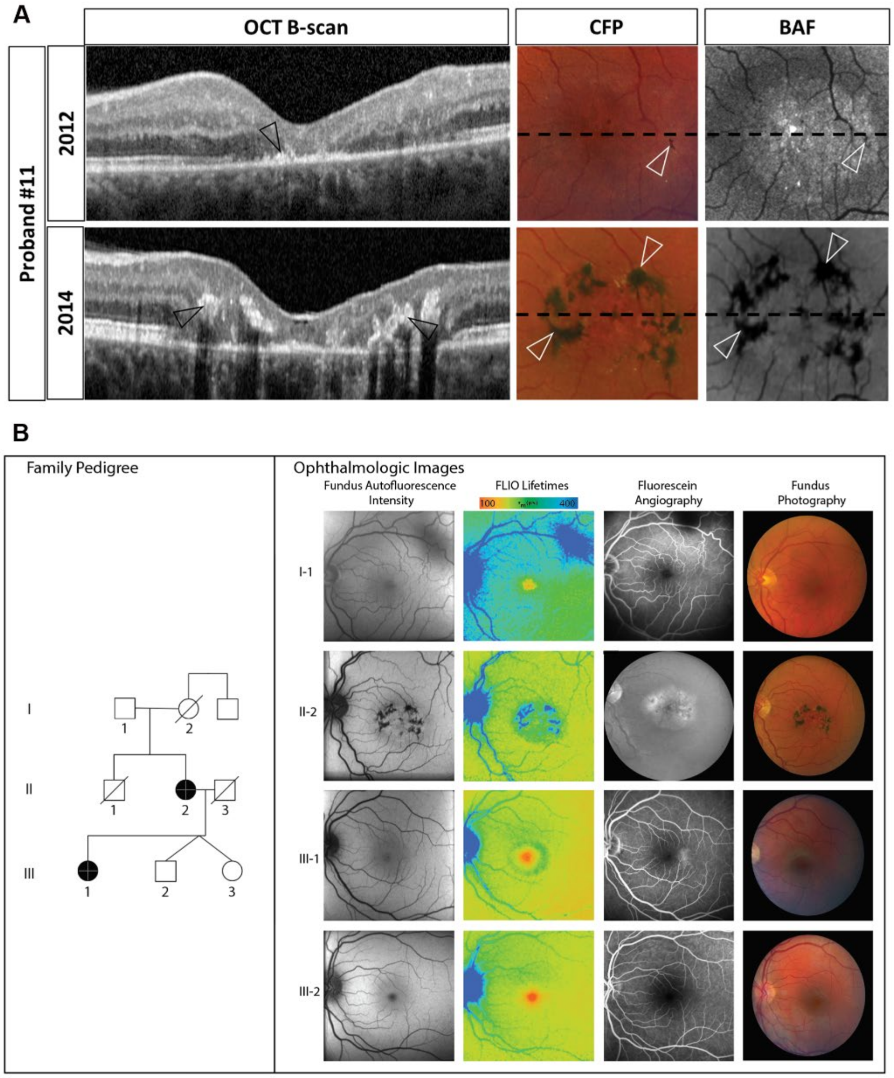

Supplemental Figure 1. Multimodal retinal imaging illustrating a fast progression of retinal changes in Donor 11 (left eye) within 33 months at an unusually young age. Both eyes showed an increase in perivascular pigment plaques (white arrow heads, visible on color fundus photographs (CFP) and blue-wavelength fundus autofluorescence (BAF) images) correspondent to hyper-reflective retinal lesions with shadowing effects (black arrow heads), as well as a progressive atrophy of outer retinal layers on optical coherence tomography (OCT) scans. Black-dotted lines indicate the position of the respective B-scans on OCT. B) Family pedigree of Donor 11 (II-2) including corresponding images of family members.

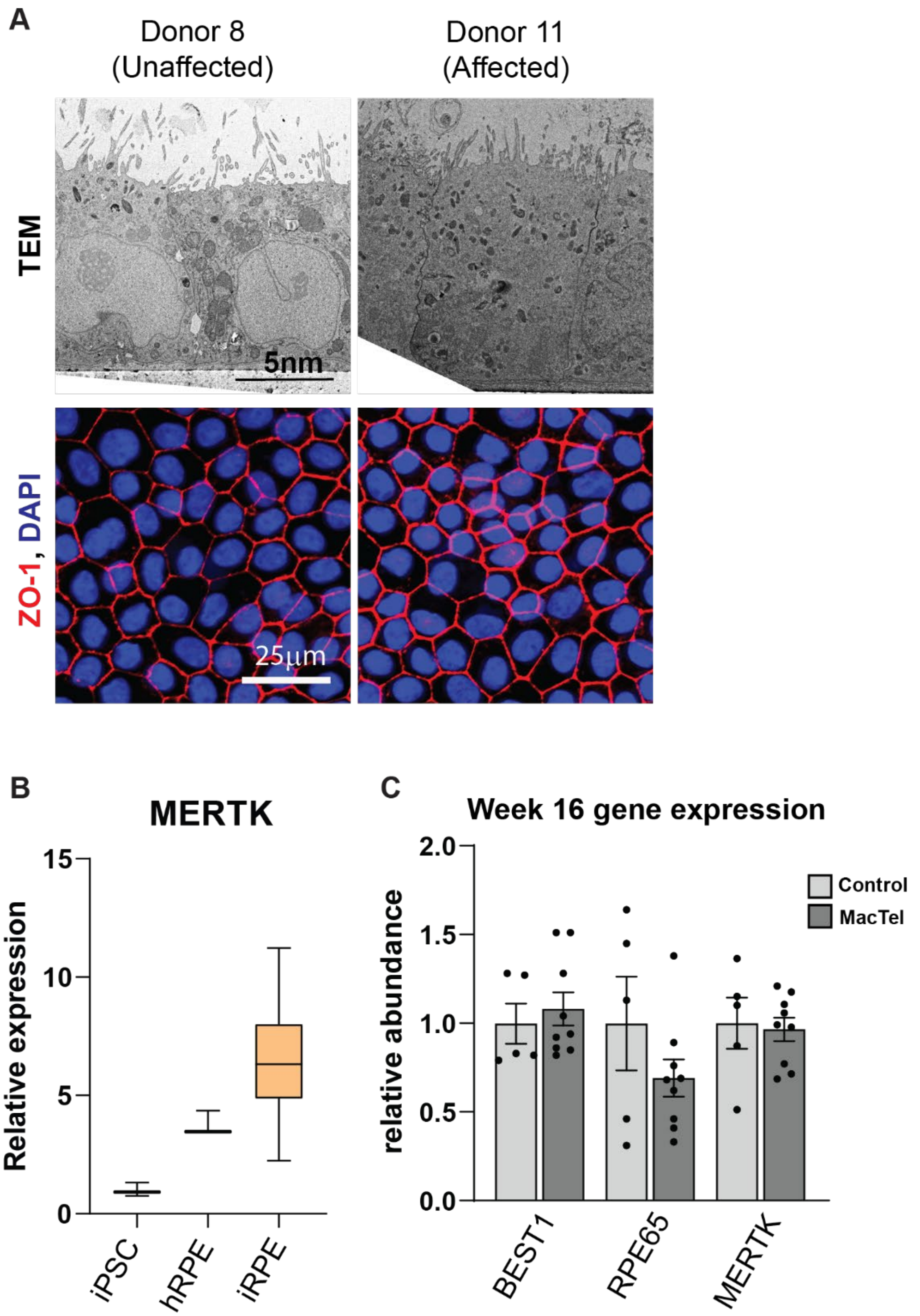

Supplemental Figure 2. A) Representative transmission electron microscopy image of iRPE cross section (upper panels) and representative iRPE confocal images of ZO-1 staining (red) and DAPI (blue) (lower panels) from unaffected and severe donor iRPE. B) Relative expression levels of RPE-specific gene MERTK in iPSCs (n=3 clones), human fetal RPE (n=3 replicates), and donor-derived iRPE (n=38 clones). Data represented as mean +/- SEM. C) Relative expression levels of BEST1, RPE65, and MERTK between unaffected (control) and affected (MacTel) donor iRPE. Data represented as mean +/- SEM. Mixed linear modeling showed no statistical differences. Control n=5, MacTel n=9 donors. Each individual donor is represented by the average of at least two independent clones.

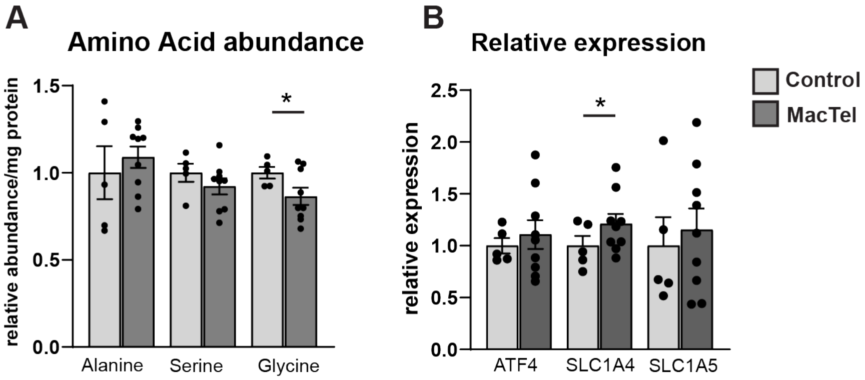

Supplemental Figure 3. A) Repeat experiment of amino acid abundance secretion. Relative abundance of alanine, serine, and glycine secreted into the media from control (n=5 donors) and MacTel (n=9 donors) iRPE. B) Relative expression of transcription factor ATF4 and amino acid transporters SLC1A4 and SLC1A5 from control (n=5 donors) and MacTel (n=9 donors) iRPE in AA deplete media. Data represented as mean of donors +/- SEM. Each individual donor is the average of independent clones assayed with technical replicates. *p<0.05 determined using mixed linear modeling.

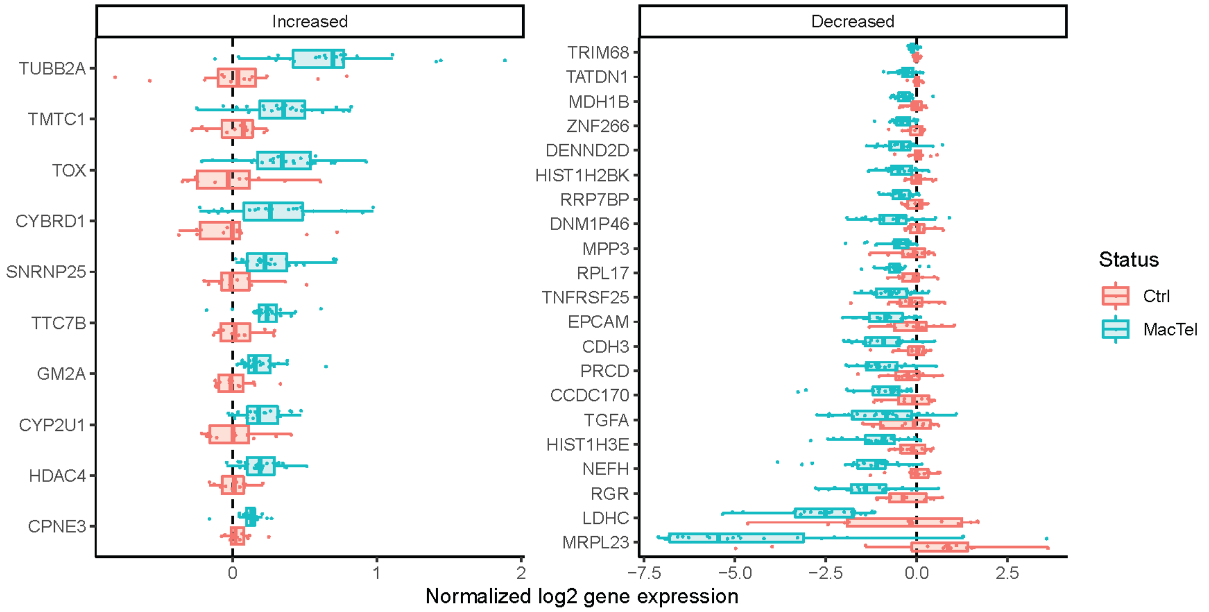

Supplemental Figure 4. Differentially expressed genes from RNAseq in all MacTel and all control iRPE. Points represent the log expression of individual clones scaled to the mean of control samples (i.e. 0), summarized in box plots where box limits (IQR) contain 50% of data, the central line indicates the median, and whiskers capture up to 1.5x IQR beyond which are the outliers.

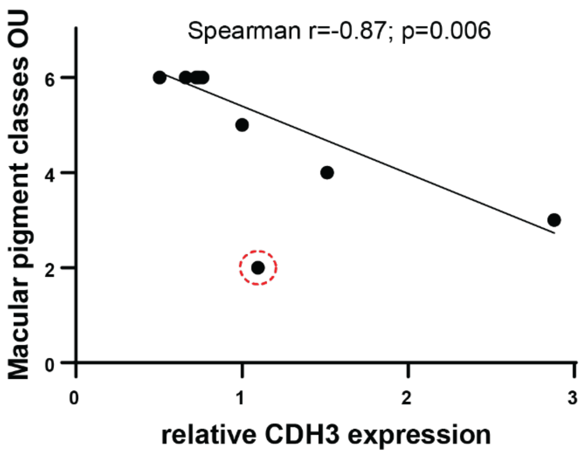

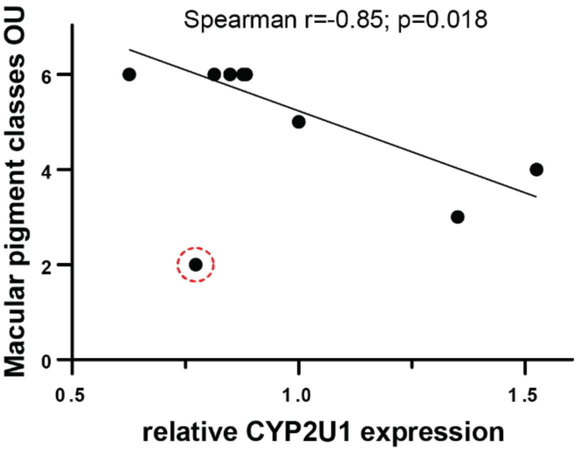

Supplemental Figure 5: Negative correlation of CDH3 and CYP2U1 expression with macular pigment classes in donors with MacTel. Correlation coefficients and p-values have been calculated excluding proband #14 (indicated with a red-dotted circle) who showed a comorbidity of MacTel and age-related macular degeneration that impacted the evaluation of the retinal phenotype. Gene expression levels were determined by qPCR. OU: both eyes.

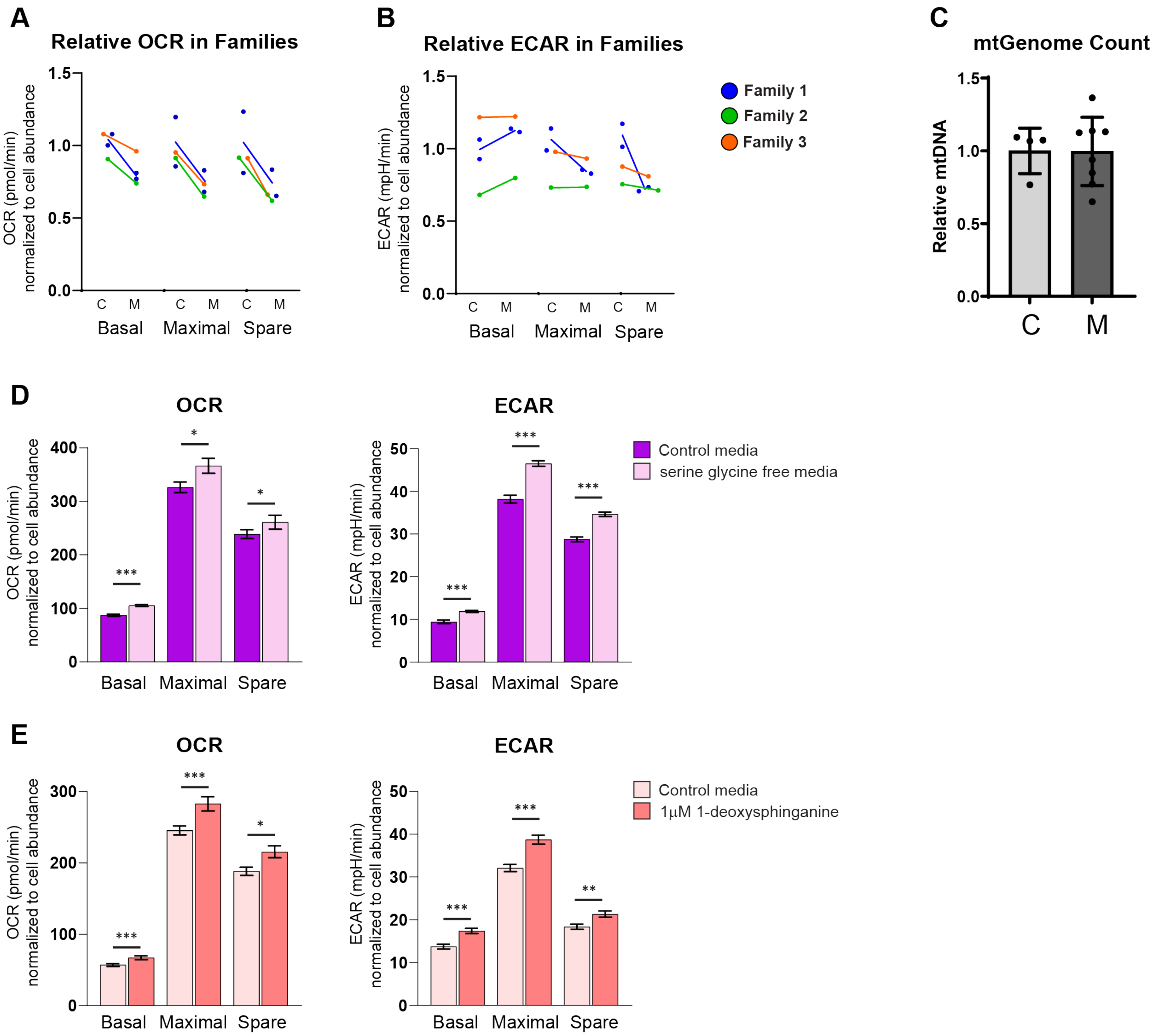

Supplemental Figure 6. Bioenergetic analysis of donors within family groups. Lines represent the change in average OCR (A) and ECAR (B) between family members with MacTel (M) and without MacTel (C). C) Relative mitochondrial genome counts in donor iRPE. Data represented as the mean of donors +/- SEM. Each individual donor is the average of at least two independent clones each run in 15-22 technical replicates. D) OCR and ECAR between control iRPE cultured in control media or media with no serine or glycine (serine glycine free media) for one week. E) OCR and ECAR of control iRPE treated with 1μM 1-deoxysphinganine for one week.

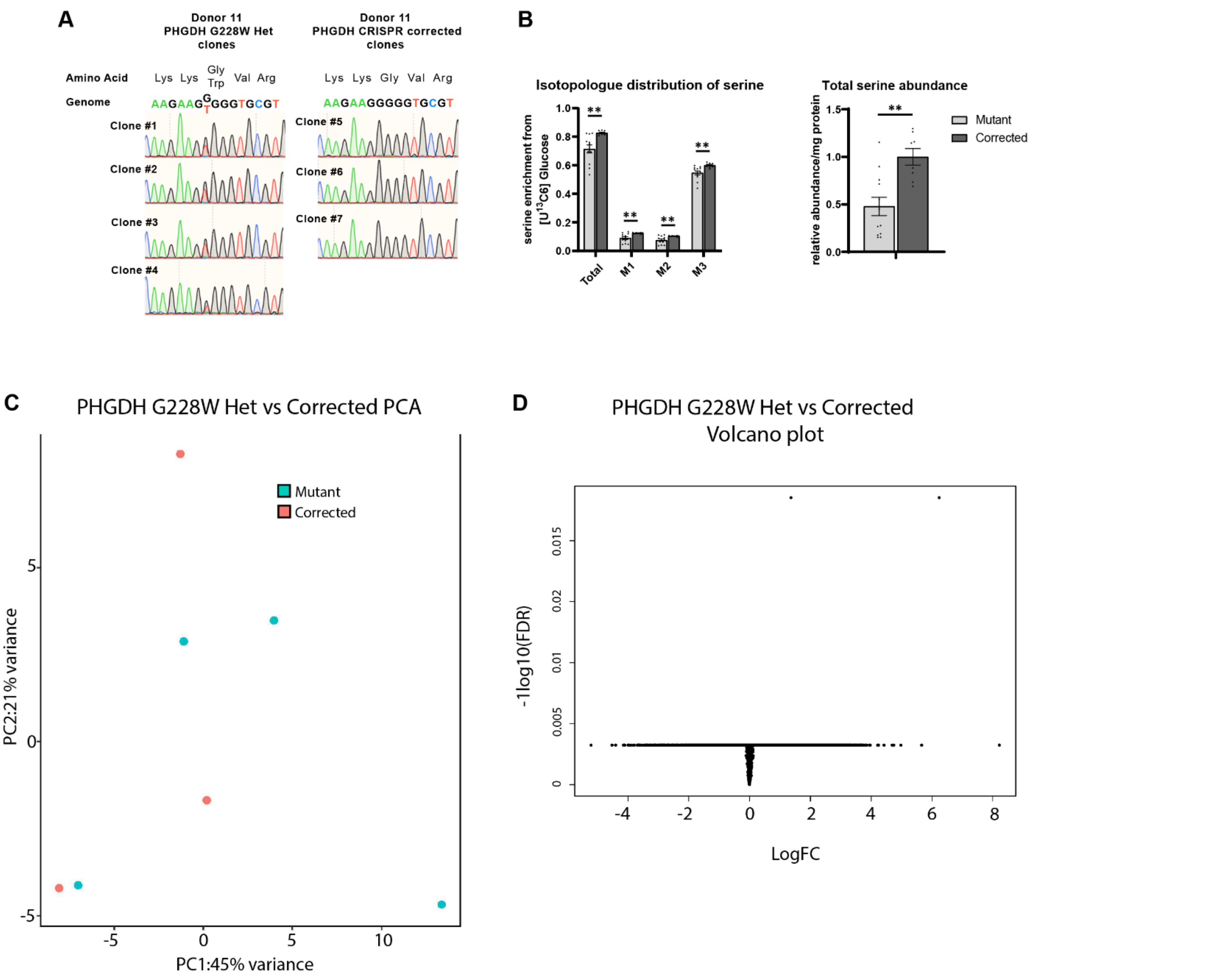

Supplemental Figure 7. A) Sequencing chromatographs of individual iPSC clones derived from donor 11 that were either successfully corrected for the PHGDH p.G228W mutation or not. B) Isotopologue distribution of U-^{13}C from glucose in serine showing the relative abundance of ^{13}C isotope in total serine, fully labeled serine (M3), and partially labeled serine (M1 and M2) from [U-$^{13}C_6$] glucose in cell culture media between donor 11 PHGDH G228W het (mutant) or PHGDH wildtype corrected (corrected) iRPE over a period of 24 hours (left panel). Relative metabolite abundance of serine in iRPE media from donor 11 PHGDH G228W het (mutant) or PHGDH wildtype corrected (corrected) iRPE (right panel). Data represented as mean +/- SEM. Individual data points are from 4 (mutant) or 3 (corrected) iRPE clones replicated 2-3 times. **$p<0.01$, statistical significance determined by student's t test. C) Principal component analysis (PCA) between individual clones of donor 11 PHGDH G228W het (mutant) or PHGDH wildtype corrected (corrected) iRPE. Dots represent individual clones. Clones from both genetic backgrounds are confounded. D) Volcano plot depicting differential expression of all genes between donor 11 PHGDH G228W het (mutant, n=4) or PHGDH wildtype corrected (corrected, n=3) iRPE. No genes have an FDR below 0.05.

Supplemental Table 1. Coding variants in MacTel-linked genes and genes examined in this study. AF<0.01

Donor	CDH3	CHCHD2	CYP2U1	G6PD	GM2A	MYC	ND4	ND5	PGAM1	PGK1	PHGDH	PRCD	PSAT	PSPH	RGR	SHMT1	SHMT2	SLC1A4	SPTLC1	SPTLC2	SPTLC3
Donor 1																					
Donor 2																					
Donor 3															p.M199V						
Donor 4															p.M199V						
Donor 5																					
Donor 6														p.T152I							
Donor 7																					
Donor 8																					
Donor 9																					
Donor 10																					
Donor 11											p.G228W										
Donor 12																					
Donor 13																					
Donor 14																					

Supplemental Table 2. Significant HALLMARK and KEGG GSEA pathways for All MacTel vs All Control donors

pathway	padj	NES
HALLMARK_CHOLESTEROL_HOMEOSTASIS	1.6E-07	2.42
HALLMARK_EPITHELIAL_MESENCHYMAL_TRANSITION	2.7E-10	2.36
HALLMARK_COAGULATION	1.6E-07	2.34
KEGG_ECM_RECEPTOR_INTERACTION	5.8E-04	2.16
KEGG_FOCAL_ADHESION	6.2E-06	2.10
HALLMARK_APOPTOSIS	1.4E-06	2.04
HALLMARK_MYOGENESIS	7.1E-07	2.04
HALLMARK_HYPOXIA	1.4E-06	2.02
HALLMARK_TNFA_SIGNALING_VIA_NFKB	2.2E-06	2.00
KEGG_GLYCOLYSIS_GLUCONEOGENESIS	7.7E-03	1.95
KEGG_GAP_JUNCTION	3.8E-03	1.93
KEGG_VIBRIO_CHOLERAE_INFECTION	9.8E-03	1.90
KEGG_PATHOGENIC_ESCHERICHIA_COLI_INFECTION	1.1E-02	1.88
KEGG_JAK_STAT_SIGNALING_PATHWAY	3.8E-03	1.88
HALLMARK_ADIPOGENESIS	6.2E-06	1.87
KEGG_GLYCOSAMINOGLYCAN_BIOSYNTHESIS_HEPARAN_SULFATE	3.7E-02	1.87
KEGG_DILATED_CARDIOMYOPATHY	9.8E-03	1.86
HALLMARK_XENOBIOTIC_METABOLISM	7.5E-05	1.86
KEGG_PROGESTERONE_MEDIATED_OOCYTE_MATURATION	9.8E-03	1.86
HALLMARK_COMPLEMENT	7.5E-05	1.84
HALLMARK_IL6_JAK_STAT3_SIGNALING	3.6E-03	1.83
KEGG_FRUCTOSE_AND_MANNOSE_METABOLISM	3.6E-02	1.83
KEGG_ARRHYTHMOGENIC_RIGHT_VENTRICULAR_CARDIOMYOPATHY_ARVC	1.4E-02	1.83
KEGG_ARACHIDONIC_ACID_METABOLISM	3.8E-02	1.79
KEGG_EPITHELIAL_CELL_SIGNALING_IN_HELICOBACTER_PYLORI_INFECTION	1.5E-02	1.79
HALLMARK_REACTIVE_OXYGEN_SPECIES_PATHWAY	4.6E-03	1.79
HALLMARK_APICAL_JUNCTION	1.8E-04	1.78
KEGG_CHEMOKINE_SIGNALING_PATHWAY	9.8E-03	1.78
KEGG_HYPERTROPHIC_CARDIOMYOPATHY_HCM	2.1E-02	1.78
HALLMARK_APICAL_SURFACE	8.6E-03	1.77
HALLMARK_ANGIOGENESIS	1.1E-02	1.77
KEGG_LYSOSOME	7.7E-03	1.77
KEGG_ARGININE_AND_PROLINE_METABOLISM	3.7E-02	1.73
HALLMARK_INTERFERON_ALPHA_RESPONSE	3.7E-03	1.69
KEGG_OOCYTE_MEIOSIS	2.1E-02	1.67
HALLMARK_ESTROGEN_RESPONSE_LATE	6.4E-03	1.57
KEGG_REGULATION_OF_ACTIN_CYTOSKELETON	2.5E-02	1.56
HALLMARK_INTERFERON_GAMMA_RESPONSE	6.7E-03	1.53
HALLMARK_UV_RESPONSE_DN	9.8E-03	1.52
HALLMARK_PROTEIN_SECRETION	1.6E-02	1.52
HALLMARK_P53_PATHWAY	9.8E-03	1.49
HALLMARK_ESTROGEN_RESPONSE_EARLY	9.8E-03	1.49
HALLMARK_KRAS_SIGNALING_UP	1.6E-02	1.47
HALLMARK_INFLAMMATORY_RESPONSE	4.0E-02	1.45
HALLMARK_IL2_STAT5_SIGNALING	2.8E-02	1.40
HALLMARK_FATTY_ACID_METABOLISM	4.3E-02	1.37
KEGG_SPLICEOSOME	1.3E-03	-1.86
HALLMARK_MYC_TARGETS_V1	8.7E-06	-1.89
HALLMARK_MYC_TARGETS_V2	4.1E-04	-2.00
KEGG_RIBOSOME	5.1E-20	-3.16

Supplemental Table 3. The non-effect and effect alleles of MacTel associated GWAS loci in donors. This table summarizes the presence of effect alleles (bold) in Genome-wide significant MacTel loci and Suggestive-significant loci as identified and defined in Bonelli et al.(18)

			**Genome-wide significant MacTel loci (non-effect:effect)**										
Patient No	MacTel status	Family	1:120265444:A:G	1:120278072:T:G	2:65220910:T:C	2:211540507:C:A	3:27706298:T:C	3:45814094:G:A	5:87847586:T:G	7:56099352:G:A	9:15302613:A:G	10:65363166:C:T	19:8235251:A:G
Patient 1	Affected	1	**GG**	TT	T**C**	CC	TT	GG	TT	**AA**	**GG**	CC	AA
Patient 2	Affected	1	**GG**	TT	T**C**	CC	TT	GG	TT	**AA**	**GG**	CC	A**G**
Patient 3	Unaffected	1	**GG**	TT	T**C**	CC	TT	GG	TT	**AA**	**GG**	CC	AA
Patient 4	Unaffected	1											
Patient 5	Affected	2	A**G**	TT	T**C**	CC	TT	GG	TT	**AA**	A**G**	C**T**	AA
Patient 6	Unaffected	2											
Patient 7	Affected	3	**GG**	TT	TT	CC	TT	G**A**	TT	**AA**	**GG**	CC	AA
Patient 8	Unaffected	3	**GG**	TT	T**C**	CC	TT	G**A**	TT	**AA**	**GG**	CC	AA
Patient 9	Affected	4	**GG**	TT	T**C**	CC	T**C**	G**A**	TT	G**A**	**GG**	CC	AA
Patient 10	Unaffected	4	**GG**	TT	T**C**	**AA**	T**C**	G**A**	TT	**AA**	**GG**	CC	AA
Patient 11	Affected		**GG**	TT	TT	CC	TT	GG	T**G**	**AA**	**GG**	CC	AA
Patient 12	Affected												
Patient 13	Affected		AA	TT	TT	CC	T**C**	GG	T**G**	GG	A**G**	CC	AA
Patient 14	Affected												

Supplemental Table 4. Significant HALLMARK and KEGG GSEA pathways for Donor 11 vs All Control donors

pathway	padj	NES
TNFA_SIGNALING_VIA_NFKB__HALLMARK	5.0E-11	2.33
COMPLEMENT_AND_COAGULATION_CASCADES_KEGG	1.9E-03	2.11
ARACHIDONIC_ACID_METABOLISM_KEGG	1.0E-03	2.10
ECM_RECEPTOR_INTERACTION_KEGG	1.0E-03	2.03
KEGG_GRAFT_VERSUS_HOST_DISEASE	3.0E-02	1.83
HALLMARK_CHOLESTEROL_HOMEOSTASIS	3.3E-03	1.80
KEGG_LINOLEIC_ACID_METABOLISM	4.1E-02	1.77
KEGG_JAK_STAT_SIGNALING_PATHWAY	2.2E-02	1.71
KEGG_ADIPOCYTOKINE_SIGNALING_PATHWAY	4.2E-02	1.71
HYPOXIA_HALLMARK	8.8E-04	1.71
HALLMARK_IL6_JAK_STAT3_SIGNALING	2.0E-02	1.65
HALLMARK_APOPTOSIS	3.5E-03	1.65
HALLMARK_INFLAMMATORY_RESPONSE	1.6E-02	1.55
HALLMARK_KRAS_SIGNALING_UP	1.3E-02	1.54
HALLMARK_EPITHELIAL_MESENCHYMAL_TRANSITION	1.3E-02	1.50
HALLMARK_MYOGENESIS	1.5E-02	1.49
HALLMARK_ADIPOGENESIS	5.9E-03	-1.50
HALLMARK_FATTY_ACID_METABOLISM	1.5E-02	-1.52
HALLMARK_MTORC1_SIGNALING	2.9E-03	-1.59
KEGG_RNA_POLYMERASE	4.2E-02	-1.74
KEGG_PROTEIN_EXPORT	2.7E-02	-1.86
KEGG_VALINE_LEUCINE_AND_ISOLEUCINE_DEGRADATION	1.1E-02	-1.88
HALLMARK_UNFOLDED_PROTEIN_RESPONSE	1.7E-04	-1.88
HALLMARK_DNA_REPAIR	6.6E-05	-1.89
HUNTINGTONS_DISEASE_KEGG	1.9E-05	-1.94
ALZHEIMERS_DISEASE_KEGG	7.0E-06	-2.07
KEGG_AMINOACYL_TRNA_BIOSYNTHESIS	8.7E-04	-2.15
KEGG_PROTEASOME	1.7E-04	-2.19
SPLICEOSOME_KEGG	1.0E-06	-2.23
MYC_TARGETS_V2_HALLMARK	4.0E-06	-2.27
CITRATE_CYCLE_TCA_CYCLE_KEGG	7.0E-06	-2.41
OXIDATIVE_PHOSPHORYLATION_KEGG	2.2E-09	-2.47
PARKINSONS_DISEASE_KEGG	3.0E-10	-2.55
MYC_TARGETS_V1_HALLMARK	5.1E-24	-2.94
OXIDATIVE_PHOSPHORYLATION_HALLMARK	3.7E-25	-3.04
RIBOSOME_KEGG	4.2E-25	-3.30

Supplemental Table 5: qPCR primer list

BEST1	Forward	CTGGGCTTCTACGTGACGC
	Reverse	TTGCTCGTCCTTGCCTTCG
RPE65	Forward	CCTGCTGGTGGTTACAAGAAA
	Reverse	CCTGCCTGTTACATGAGCTGT
PSPH	Forward	GAGGACGCGGTGTCAGAAAT
	Reverse	GGTTGCTCTGCTATGAGTCTCT
SHMT1	Forward	CTGGCACAACCCCTCAAAGA
	Reverse	AGGCAATCAGCTCCAATCCAA
SHMT2	Forward	CCCTTCTGCAACCTCACGAC
	Reverse	TGAGCTTATAGGGCATAGACTCG
PHGDH	Forward	CTGCGGAAAGTGCTCATCAGT
	Reverse	TGGCAGAGCGAACAATAAGGC
PSAT1	Forward	TGCCGCACTCAGTGTTGTTAG
	Reverse	GCAATTCCCGCACAAGATTCT
ATF4	Forward	ATGACCGAAATGAGCTTCCTG
	Reverse	GCTGGAGAACCCATGAGGT
SLC1A4	Forward	TGTTTGCTCTGGTGTTAGGAGT
	Reverse	CGCCTCGTTGAGGGAATTGAA
SLC1A5	Forward	GAGCTGCTTATCCGCTTCTTC
	Reverse	GGGGCGTACCACATGATCC
36B4	Forward	GAAGCCACGCTGCTGAACAT
	Reverse	CAAGGCCAGGACTCGTTTGTA
CYP2U1	Forward	CCTCTGGTGGGCAACTTCG

	Reverse	GCCTATGACCGAGGGATCAATC
CDH3	Forward	ATCATCGTGACCGACCAGAAT
	Reverse	GACTCCCTCTAAGACACTCCC
PRCD	Forward	TCCTCAGGCAGGGAGAAAGA
	Reverse	ATTTCAGCAGAGCAGCCAG
GM2A	Forward	AGCCATCCCAGCTCAGTAG
	Reverse	GGCTCCCCAGTAGGAATTAACA
RGR	Forward	GTTGGGGTCACTACGACTATGA
	Reverse	GGCATGGCGAAGTTGAAGAA
